# RNA-sequencing based gene expression landscape of guava *cv*. Allahabad Safeda and comparative analysis to colored cultivars

**DOI:** 10.1186/s12864-020-06883-6

**Published:** 2020-07-15

**Authors:** Amandeep Mittal, Inderjit Singh Yadav, Naresh Kumar Arora, Rajbir Singh Boora, Meenakshi Mittal, Parwinder Kaur, William Erskine, Parveen Chhuneja, Manav Indra Singh Gill, Kuldeep Singh

**Affiliations:** 1 School of Agricultural Biotechnology, Punjab Agricultural University, Ludhiana, Punjab 141004 India; 2 Department of Fruit Science, Punjab Agricultural University, Ludhiana, 141004 India; 3 Fruit Research Sub-Station, Punjab Agricultural University, Bahadurgarh, Patiala, 147002 India; 4 Plant Breeding and Genetics, Punjab Agricultural University, Ludhiana, Punjab 141004 India; 5 Centre for Plant Genetics and Breeding, University of Western Australia, Perth, WA 6907 Australia; 6 ICAR-National Bureau of Plant Genetics Resources, New Delhi, 110012 India

**Keywords:** Guava RNA-Seq, Fruit development and ripening, Allahabad Safeda, Punjab Pink, Apple Color, Secondary metabolites, Candidate genes for fruit color

## Abstract

**Background:**

Guava (*Psidium guajava* L.) is an important fruit crop of tropical and subtropical areas of the world. Genomics resources in guava are scanty. RNA-Seq based tissue specific expressed genomic information, de novo transcriptome assembly, functional annotation and differential expression among contrasting genotypes has a potential to set the stage for the functional genomics for traits of commerce like colored flesh and apple color peel.

**Results:**

Development of fruit from flower involves orchestration of myriad molecular switches. We did comparative transcriptome sequencing on leaf, flower and fruit tissues of *cv.* Allahabad Safeda to understand important genes and pathways controlling fruit development. Tissue specific RNA sequencing and de novo transcriptome assembly using Trinity pipeline provided us the first reference transcriptome for guava consisting of 84,206 genes comprising 279,792 total transcripts with a N50 of 3603 bp. Blast2GO assigned annotation to 116,629 transcripts and PFam based HMM profile annotated 140,061 transcripts with protein domains. Differential expression with EdgeR identified 3033 genes in Allahabad Safeda tissues. Mapping the differentially expressed transcripts over molecular pathways indicate significant Ethylene and Abscisic acid hormonal changes and secondary metabolites, carbohydrate metabolism and fruit softening related gene transcripts during fruit development, maturation and ripening. Differential expression analysis among colored tissue comparisons in 3 cultivars Allahabad Safeda, Punjab Pink and Apple Color identified 68 candidate genes that might be controlling color development in guava fruit. Comparisons of red vs green peel in Apple Color, white pulp vs red pulp in Punjab Pink and fruit maturation vs ripening in non-colored Allahabad Safeda indicates up-regulation of ethylene biosynthesis accompanied to secondary metabolism like phenylpropanoid and monolignol pathways.

**Conclusions:**

Benchmarking Universal Single-Copy Orthologs analysis of de novo transcriptome of guava with eudicots identified 93.7% complete BUSCO genes. In silico differential gene expression among tissue types of Allahabad Safeda and validation of candidate genes with qRT-PCR in contrasting color genotypes promises the utility of this first guava transcriptome for its potential of tapping the genetic elements from germplasm collections for enhancing fruit traits.

## Background

Guava (*Psidium guajava* L.) fruit is a berry with edible pericarp tissue as flesh and has excellent antioxidant properties [1]. Guava is member of family Myrtaceae (possesses ~ 150 species) and has 2n = 22 chromosomes with a genome size of ~ 450 MB [2, 3]. Guava popularly known as ‘Apple of the Tropics’ is a native of tropical America from where it was distributed in all tropical and subtropical areas of the world [4, 5]. India, Mexico, Pakistan, Taiwan, Thailand, Colombia, Indonesia are major producers of guava and a small-scale plantation is done in Malaysia, Australia and South Africa [6].

Fruiting branches in guava bear three terminal flower buds and the central floral bud develop faster into fruit compared to other two lateral buds. In Northern India subtropics, there are two flowering seasons *viz*. April–May and August – September with peak anthesis time of flower bud between 5:00–7:30 AM. Guava flowers are hermaphrodite and carry 160–400 bilobed anthers and an ovary which is inferior, syncarpous with axile placentation and subulate terminal style [6]. Style being longer than filaments, self-pollination is less common and domestic honeybee (*Apis mellifera*) is the chief pollinator [7]. There are more than 400 guava cultivars grown around the world with variation in fruit pulp and peel color. Fruit pulp color ranges from white to deep pink and fruit skin turns green to yellow or red upon ripening and this character varies among cultivars and depends upon the season [7].

Guava is India’s fourth most important fruit crop after mango, banana, citrus and is popularly known as poor man’s apple because of low cultivation cost and high nutritive value. Guava is a climacteric fruit and contains reducing sugars, indigestible lignin fiber and carotenoids that increase as the fruit ripens [8] with major cell wall hydrolyzing enzymes like polygalacturonases, cellulases and starch hydrolyzing α-, β-amylases [9]. Guava possesses large quantities of vitamin C [6], is a rich source of phenolic compounds [10] and carries secondary metabolites with medicinal properties [11, 12]. Guava intake induces resistance against infectious agents such as *Staphyloccocus*, scavenge cancer causing free radicals and helps in the structural protein, collagen synthesis which maintains integrity of blood vessels, skin, organs, and bones [13].

Colored fruits are preferred by the consumer owing to higher nutraceutical properties. Color in fruits and vegetables are controlled by secondary metabolism pathway genes mainly phenylalanine ammonia-lyase (PAL), chalcone synthase (CHS), dihydro- flavonol 4-reductase (DFR), flavanol synthase/flavanone 3-hydroxylase (F3H), UDP-glucose:flavonoid 3-O-glucosyltransferase (UFGT), anthocyanidin synthase (ANS) and transcription factors (TFs) of myeloblastosis (MYB), basic helix-loop-helix (bHLH), tryptophan- aspartic acid (WD) repeats, NAC (NAM, ATAF1/2 and CUC2) and MADS (MCM1, AGAMOUS, DEFICIENS, and SRF) domain [14–19]. For an instance, expression of genes encoding MYB TFs, 4-coumarate-CoA ligase (4CL), Glutathione S transferase (GST), Flavonoid 3′5’ hydroxylase (F3’5’H) and WD repeat are expressed at higher levels in the red-fleshed apples compared with green apples in congruence with the higher levels of flavonoid and anthocyanin accumulation in red-fleshed apples [20]. MADS18 is implicated in regulation of anthocyanin synthesis in red compared to green pear [21] and a NAC TF named as BLOOD makes a heterodimer with PpNAC1 up-regulating the MYB TFs leading to anthocyanin accumulation in blood-fleshed peach [22]. Also, 32 red peel-color-related genes have been identified in Longan together with anthocyanin biosynthesis genes [23]. However, in red-fleshed orange ‘Hong Anliu’, lycopene accumulation is the primary cause behind flesh color [24]. Also, a green tomato inbred line BUC30 have mutations in phytoenesynthetase1 (PSY1), STAY-GREEN (SGR), and SlMYB12 genes leading to no carotenoids and no degradation of chlorophylls in green ripe tomatoes compared to KNR3 red-fruits [25].

No such studies have so far been conducted in guava. Also, there exists enormous gene sequence variation among species that generating consensus sequence-based markers and validation is labor intensive and non-targeted. Developing new colored genotypes with desirable agronomic traits by hybridization without marker assisted selection for color related genes is a time-consuming process. So, generating expressed genic sequence information at genome wide level is important to expedite gene cloning and tapping in color trait controlling loci from agronomically less preferred colored guava cultivars (owing to low yields and/or lesser shelf life). Tissue specific comparative gene expression within a genotype and comparison to contrasting genotypes by RNA-Seq is an alternate targeted approach in the absence of gold standard genome assembly.

To generate a global gene expression landscape in guava we generated RNA-Seq libraries from leaf, flower buds and fruit tissue of green skinned/white pulped table purpose guava cv. Allahabad Safeda (AS). In another cv. Apple Color (AC) fruit peel color changes from green to apple color (reddish) at fruit picking stage and peel becomes leathery within 3–5 days in winter season. Pink pulp cv. Punjab Pink (PP) is commercially grown for red nectar and the color develops during maturation process (immature fruits have white pulp) probably owing to the chromoplast development as found in other similar genotypes [26]. Comparative RNA-seq of leaf, flower and fruit at various developmental stages of AS, red vs green peel of AC and pink pulp of PP vs white pulp of AS in current study enhances our understanding of color development in guava and identifying important color controlling candidate genes. Most importantly this study provides the first de novo transcriptome of guava setting a stage for guava genomics at genome wide scale.

## Results

**Fig. 1:**
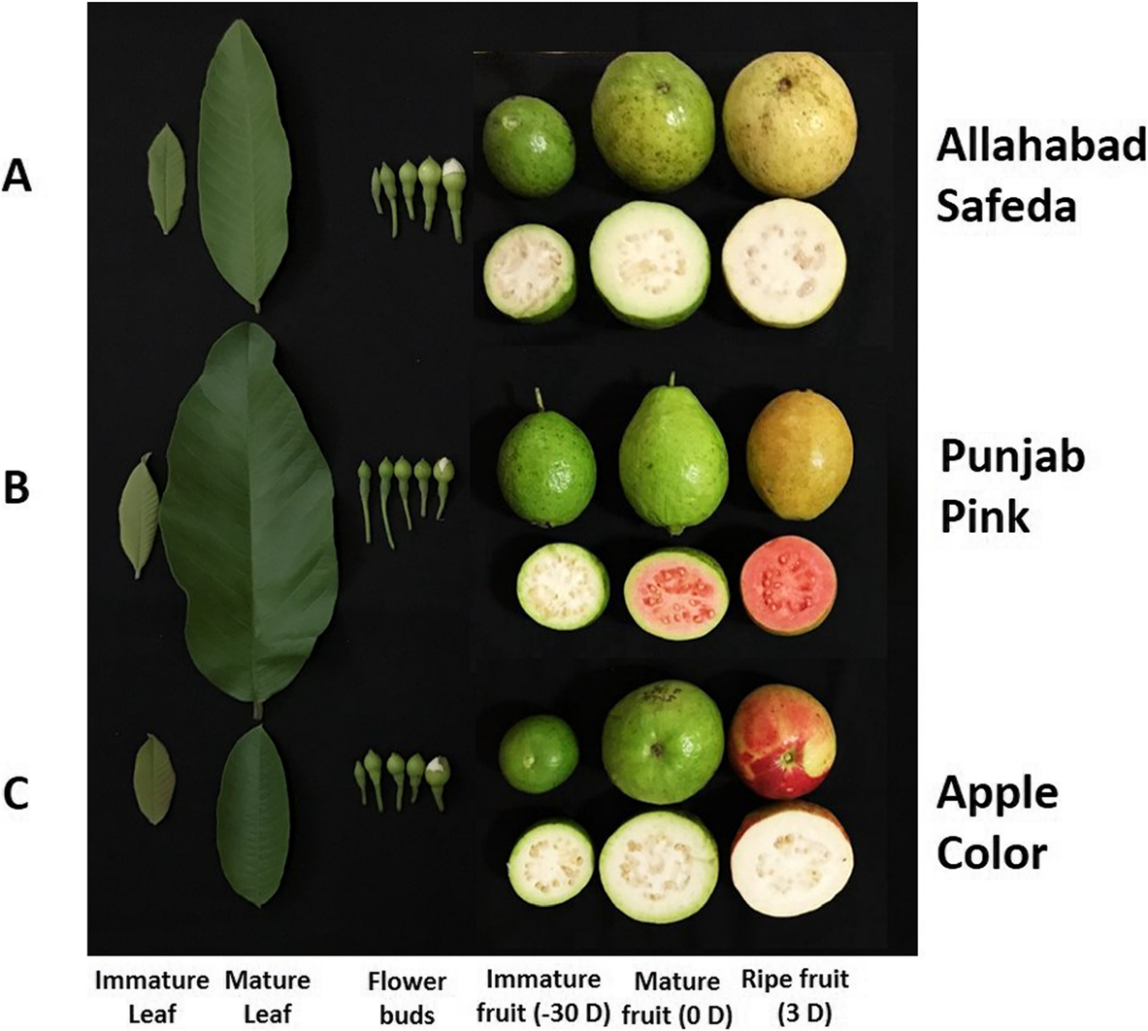
Leaves, flower buds and fruits color comparison in Allahabad Safeda, Punjab Pink and Apple Color - CISH G5 genotypes of guava

**Fig. 2:**
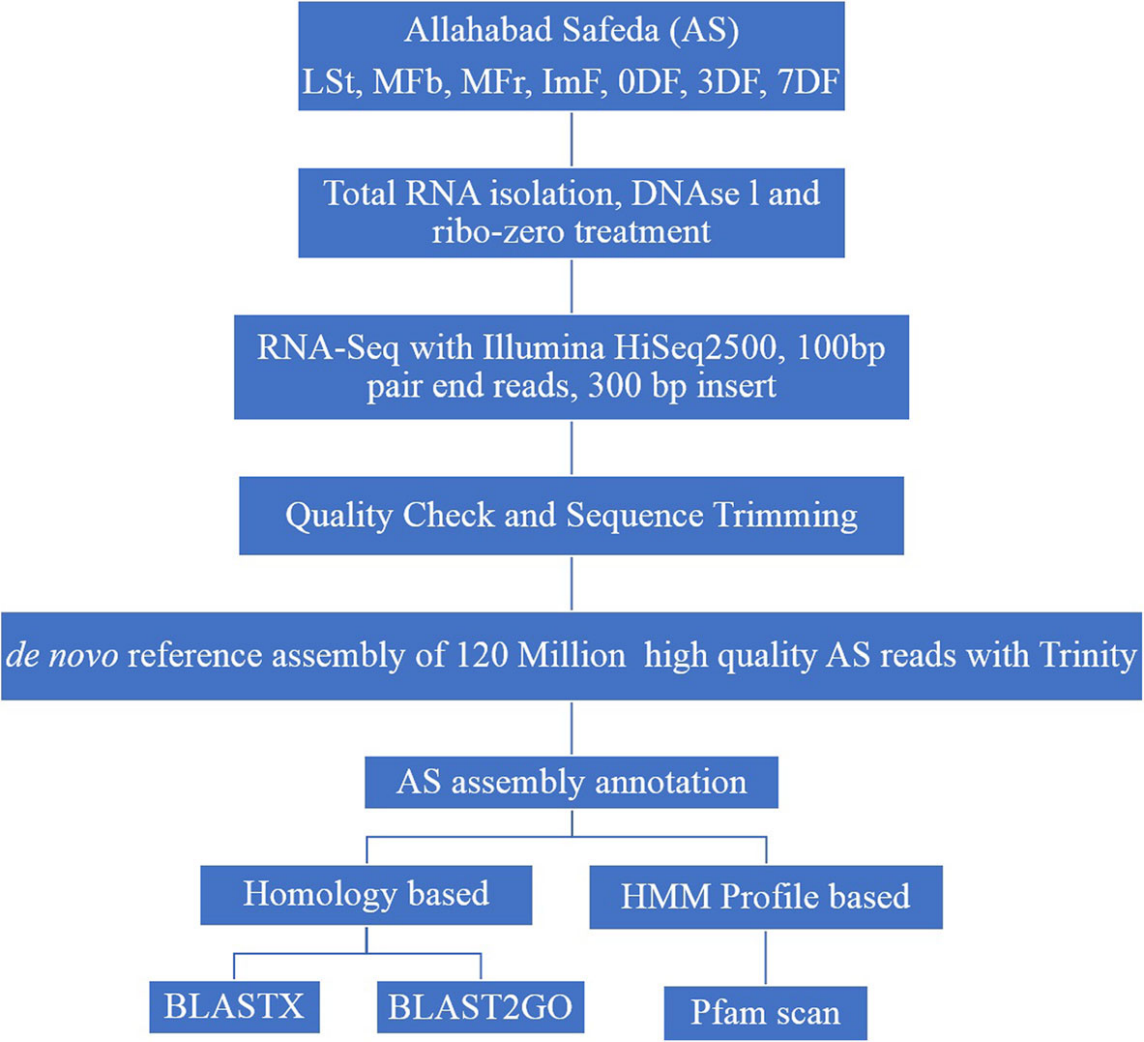
Experimental set up of de novo transcriptome assembly of *Psidium guajava* L. *cv.* Allahabad Safeda and functional annotation of transcripts. LSt – Leaf and Shoot tip tissue (Immature leaf, Mature leaf and shoot apex), MFb – Mixed Flower bud tissue (six developmental stages), MFr – Mixed Fruit tissue (Immature, just harvested, 3 days ripe and 7 days ripe fruit – with seed and peel), ImF – Immature Fruit (80 day before harvesting, without seed), 0DF – Zero Day Fruit (Mature just harvested, without seed), 3DF – Three Days after harvesting Fruit (ripe fruit, without seed), 7DF – Seven Day after harvesting Fruit (over-ripe fruit, without seed)

We have developed the first de novo reference transcriptome assembly of guava, performed gene annotations, compared different fruit development stages to understand molecular pathway (s) in fruit ripening and compared 3 different genotypes with variable coloration in pulp and fruit skin/peel to understand the fruit color development pathway in guava (Fig. 1 & Fig. 2). Allahabad Safeda (AS) is the widely grown table purpose guava cultivar of India and has green foliage. Figure 1a shows that the floral buds at all the growth stages of AS are green in color, and exhibits white colored petals as flower opens. Immature and mature fruits of AS both have white pulp and green skin. During ripening fruit skin turns yellow within 3 days after harvesting and stays yellow thereafter. Punjab Pink (PP) has darker green foliage (Fig. 1b) and floral buds compared to AS. Although pulp color in PP is white in immature fruit but turns pink in mature fruit (Fig. 1b). Apple Color (AC) has green foliage, green floral buds, white flowers and white pulp of immature and mature fruits but the skin of fruit changes its color from green to crimson red (apple color) at maturation within 3–5 days in winter season (Fig. 1c). We have compared the RNA-Seq (methods) of Allahabad Safeda leaf and shoot tip (LSt), mixed flower buds (MFb) and mixed fruits (MFr) to understand the landscape of molecular changes in fruit development of guava. We have also compared the immature (ImF), mature (0DF), ripe (3DF), and over-ripe (7DF) fruit growth stages to understand maturation and ripening of guava fruit. To identify inducible genes resulting into apple color development in colored genotypes, we compared red vs green skin of AC and mature fruit of AS to PP.

### RNA-Seq data generation, de novo transcriptome assembly and annotation

**Fig. 3:**
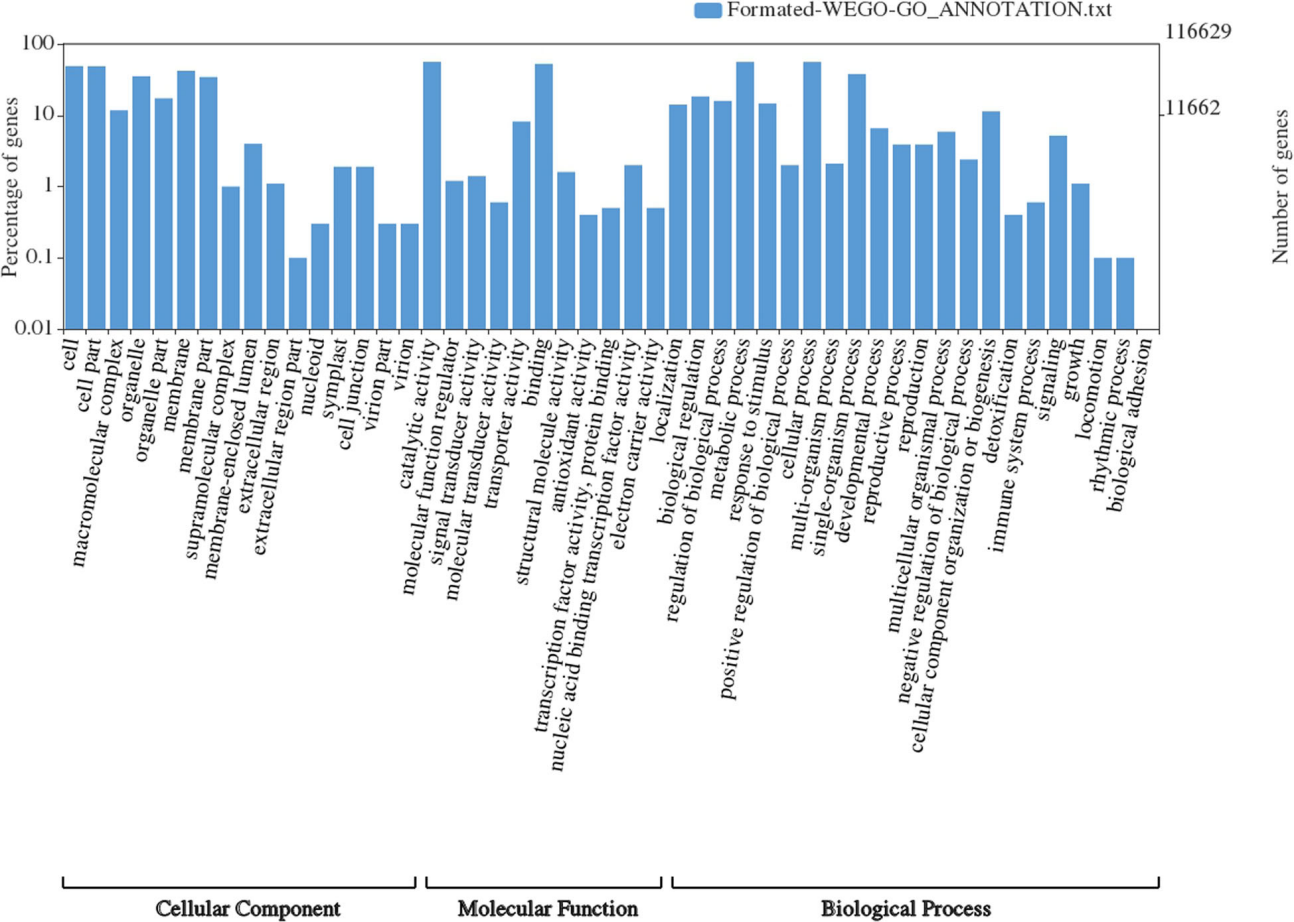
Distribution of assembled transcripts in the gene ontology classes of biological processes, molecular functions and cellular components. Bars are scaled to log values

The pair end libraries from different tissue types of AS, AC and PP were sequenced and 137.3, 20.24 and 20 million raw reads of 100 bp each were generated, respectively (Additional file 1: Table S1). The low quality sequences were filtered at quality score ≥ 30 and 120 million high quality reads of AS belonging to 13 libraries were used for generating de novo reference transcriptome using Trinity assembler [27, 28]. A total of 279,792 transcripts belonging to 84,206 components/genes with N_50_ of 3603 bp were obtained (Table 1). Benchmarking Universal Single-Copy Orthologs (BUSCO) analysis [29] with eudicots identified 93.7% (1987/2121) complete BUSCO genes, 4.5% (95) fragmented orthologs and 1.8% (39) orthologs as missing (Additional file 6: Figure S1). Blast search against the nr protein database identified homologs for 219,924 transcripts. Protein family search identified 140,061 protein family domains. Gene ontology assessment with Blast2GO assigned gene ontology terms to 116,629 transcripts (Table 1; Fig. 3; Additional file 13: Data S1), where biological process consists of 87,954 transcripts, cellular components of 82,820 and molecular function of 96,308 transcripts (Fig. 3, Additional file 7: Figure S2).

**Table 1:** Allahabad Safeda transcriptome assembly statistics

**Transcriptome Assembly**	
Contigs/Transcripts	279,792
Components/Genes	84,206
% GC content	43.08
Contig N50	3603
Assembly length (MB)	647.4
**Functional annotation**	
Transcripts with homologs	219,924
Match with predicted protein	9958
Match with hypothetical protein	7790
**Protein Family annotation**	
Transcripts with Pfam domains	140,061
**Gene Ontology Annotation**	
Transcripts with assigned GO terms	116,629
Biological Processes	87,954
Cellular Component	82,820
Molecular Function	96,308

### Differential expression analysis of leaf, flower and fruit of Allahabad Safeda

**Fig. 4:**
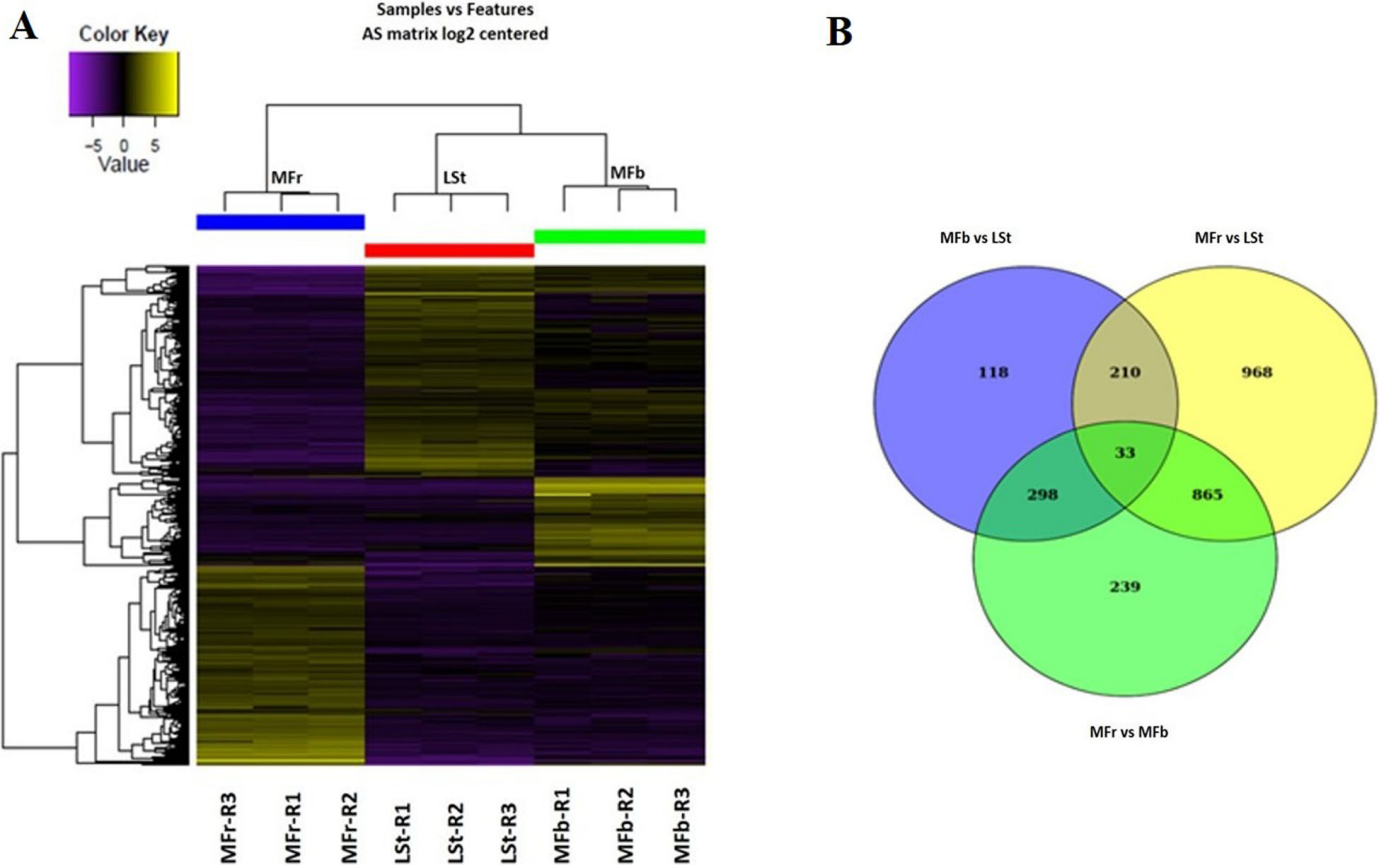
Differentially expressed transcripts in Allahabad Safeda tissues **a** Heatmap and hierarchal clustering **b** Venn diagram in tissue types viz. Leaf and shoot tip (LSt), Mixed flower buds (MFb) and Mixed fruit tissue (MFr). R1, R2 and R3 are the three RNA-Seq biological replicates

In AS 2777 transcripts representing 2139 genes were found differentially expressed in mixed fruit (MFr) vs mixed flower buds (MFb), mixed fruit vs leaf & shoot tip (LSt) and mixed flower bud vs leaf & shoot tip (Data S2). Clustering analysis shows a high correlation among the replicated samples > 0.96 for LSt, > 0.93 for MFb and > 0.97 for MFr (Fig. 4a; Additional file 2: Table S2).

We identified 2125 differentially expressed transcripts (DETs) in MFr compared to LSt, with 971 being up and 1154 down regulated. In MFr and MFb comparison, 1445 DETs were found, of which 719 were up-regulated and 726 down regulated. However, 660 DETs were identified between MFb and LSt, with 447 up and 213 down-regulated (Additional file 13: Data S2). Only 33 transcripts among the DETs were common among the three tissues types (Fig. 4b). In order to identify genes involved in fruit development, top 20 up-regulated transcripts were selected from MFr comparison to LSt and/ or MFb and 7 genes were found common with > 10 Log2FC. Interestingly, putting together transcripts of these two comparisons all were found co-upregulated and none of the genes was found down regulated (Additional file 3: Table S3).

The most up-regulated gene (comp27411_c1) represented by six transcripts Hydroxycinnamoyl CoA shikimate (quinate hydroxycinnamoyltransferase, HCT) belonging to BAHD family of acyl-CoA-dependent acyl-transferases controls lignin [30, 31] and cutin biosynthesis [32]. Cinnamyl alcohol dehydrogenase (CAD) important for lignin biosynthesis [33, 34], expansins involved in cell wall loosening [35], ABC transporter encoding ATP dependent channels [36], Palmitoyl transferase involved in fatty acid oxidation [37], 1-aminocyclopropane-1-carboxylate oxidase (ACO) an ethylene biosynthesis gene [38], Subtilisin-like protease with a role in plant-pathogen interactions [39], 9-cis-epoxycarotenoid dioxygenase (NCED) a major Abscisic Acid biosynthesis gene [40, 41] and Rbcx, a Rubisco assembly chaperon [42] are the top protein families represented by up-regulated transcripts in guava fruit (Additional file 3: Table S3).

### Metabolic pathway analysis of fruit tissue in comparison to leaf and flower

**Fig. 5:**
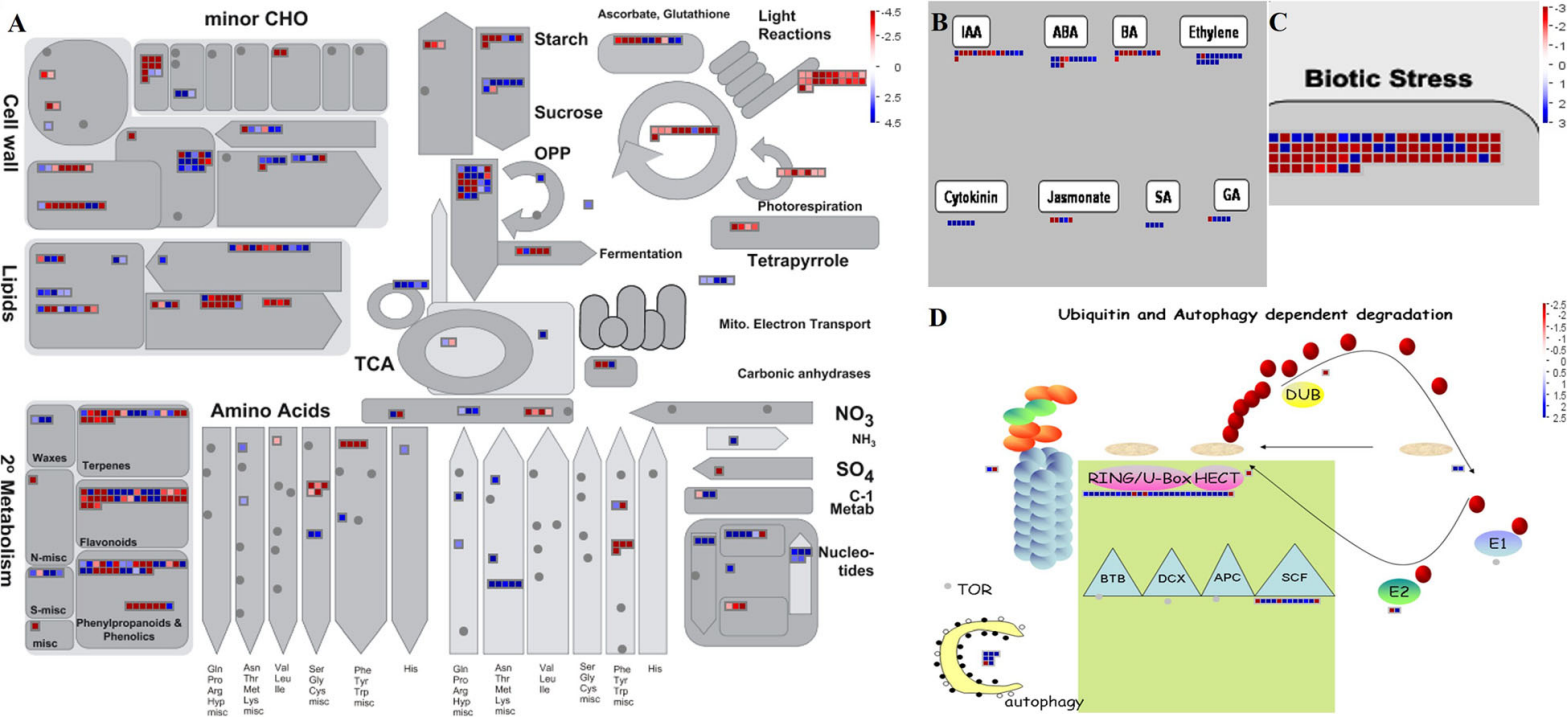
MAPMAN pathway distribution of differentially expressed transcripts of fruit (MFr) vs leaf (LSt) **a** metabolism overview **b** regulation overview **c** cellular response overview **d** proteasome and autophagy. Up- and Down- regulated DETs are represented with blue and red squares, respectively with log2 transformed values (scale for **b** and **c** is same)

The metabolic and regulatory pathway analysis of fruit, the major sink in comparison to the strongest source, the leaf was performed with MAPMAN software [43] (http://mapman.gabipd.org) with all DETs at FDR < 0.001. Differential 2125 transcripts were found significantly regulated in fruit compared to leaf (Fig. 5; Additional file 13: Data S2). General metabolism analysis showed that transcripts involved in light reactions, C3 cycle, photosynthesis, tetrapyrole pathway (controlling chlorophyll biosynthesis), starch synthesis, amino acid biosynthesis except phenylalanine (input for secondary metabolism), lipid degradation, raffinose biosynthesis, cell wall associated leucine rich repeat and arabinogalactan-proteins are down-regulated in fruit. Importantly sucrose biosynthesis, gluconeogenesis, conversion of starch to reducing sugars like glucose and fructose, wax biosynthesis, phenylalanine generation, glycolipid synthesis (for generating mono and di galactosyl diacylglycerol for food reserve storage in seeds), cellulose synthesis, trehalose biosynthesis, mitochondrial electron transport chain, cell wall degradation pectate lyases (PeLs) and polygalacturonases (PGs) are up-regulated in fruit. Discreet furcation of these pathways in fruit tissue are in general concordance with its biological role of alluring birds for seed dispersal [16]. However, pectin esterases involved in plant cell wall modification and subsequent breakdown and long chain fatty acid biosynthesis genes catalyzing the cutin synthesis exhibited a mixed response (Fig. 5a).

Regulation overview analysis with MAPMAN shows that most of the transcripts mapping to ABA, Ethylene, Cytokinin, Gibberellins (GA) and Salicylic acid (SA) signal transduction pathways were up-regulated whereas Jasmonate (JA), Auxin, and Brassinosteroid (BR) were down-regulated with few transcripts showing up-regulation (Fig. 5b). Ethylene biosynthesis and signal transduction genes, 1-aminocyclopropane-1-carboxylate synthase (ACS), ACC oxidase 3, Ethylene receptor 2 (ETR2), ethylene response factor ERF-1, basic helix-loop-helix (bHLH) TF and pyridoxine biosynthesis gene PDX1.2 were found up-regulated. ABA biosynthesis and signaling factors including NCED and ABA binding factor 4 (ABF4), B3 domain containing high-level expression of sugar-inducible gene 2 (HSI2), highly ABA-induced 1 (HAI1), hypostatin resistance 1 (HYR1), a UDP glycosyltransferase (UGT) and GRAM domain family protein were highly up-regulated, only ABA-responsive TB2/DP1 (HVA22 family protein) showed down-regulation. Auxin leucine-rich repeats (LRR), F-box TIR receptor, TCP family, ARF and AUX/IAA TFs were found down regulated indicating the auxin signaling down-regulation in guava fruit development. Interestingly, Brassinosteroid insensitive (BRI) encoding receptor kinase is up-regulated indicating overall up-regulation of BR signal transduction and responses.

We identified 40 TF families with multiple transcripts belonging to MYB, MADS, HB, WRKY, ARF, bHLH, AP/EREBP, bZIP, NAC, AUX/IAA, B3, Jumonji and, Polycomb. These families showed both up and down regulation, indicating their importance in modulation of fruit development. Sucrose cytosolic invertase 2 (CINV2), responsible for conversion of sucrose to monosaccharides like fructose and glucose showed up-regulation and is in line with increase in sucrose catabolism in developing fruits.

Cellular response analysis depicts down regulation of transcripts belonging to biotic stress and is in line with fruits being more prone to pathogen and insect damage in comparison to leaves (Fig. 5c). Phytoene synthase (PSY) and lycopene beta cyclase (lcy-b) responsible for accumulation of α and β- carotene shows over-expression indicating up-regulation of carotenoid biosynthesis pathway. Ubiquitin and autophagy dependent degradation pathways (Fig. 5d) showed up-regulation of 44 transcripts, emphasizing increased protein turnover process. Near similar results were obtained in a comparison of fruit vs flower transcripts (Additional file 13: Data S2).

### Up-regulation of secondary metabolites during fruit ripening

We compared RNA-Seq at different fruit maturity and ripening stages in AS. Comparison of mature fruit 0DF to immature fruit ImF identified 220 differentially regulated transcripts, with 75 showing up-regulation and 145 showing down regulation (Additional file 13: Data S3). However, at ripening 3DF vs 0DF, 366 transcripts were differentially regulated with 232 up-regulated and 144 down-regulated (Additional file 13: Data S4). Interestingly, during over-ripening 7DF vs 3DF only 11 transcripts showed differential regulation with only one down regulated (Additional file 13: Data S5). The major up-regulated genes in mature vs immature fruit (Additional file 8: Figure S3; Additional file 13: Data S3) include Alpha-Expansin, cellulose synthase, phospho-enol-pyruvate carboxylase kinase, β-amylase, PSY, CAD and COMT family of lignin biosynthesis genes and other o-methyl transferases. However, flavonoid pathway genes other than lignin biosynthesis, pectin methylesterases, light reactions, calvin cycle and photorespiration were down-regulated.

In ripe vs mature fruit (Additional file 9: Figure S4 A; Additional file 13: Data S4) there is upregulation of transcripts for cellulose synthase, expansins, increased fatty acid synthesis and elongation, PSY, phenylalanine biosynthesis genes arogenate dehydratase, flavonoid biosynthesis related transcripts like UGT - Hypostatin Resistance 1 (HYR1), Flavonoid 3′,5′-hydroxylase 2 (F3′5′H), Phenylalanine ammonia-lyase 3 (PAL 3) and 4-coumarate--CoA ligase 2 (4CL2). All the transcripts belonging to ABA, BR, Ethylene, Cytokinin, and SA were up-regulated, while AUX and TCP transcripts related to auxin and GA Insensitive (GAI) were down-regulated (Data S4, Figure S4 B). Transcripts corresponding to TF families of WRKY, AP2, bHLH, PHOR1 (ubiquitin ligase activity), MYB and C2C2.CO like were found up-regulated. Also, Ubiquitin and autophagy dependent protein turnover pathway were up-regulated (Data S4, Figure S4 C) as well.

### Apple color in fruit skin is derived from up-regulation of secondary metabolism

**Fig. 6:**
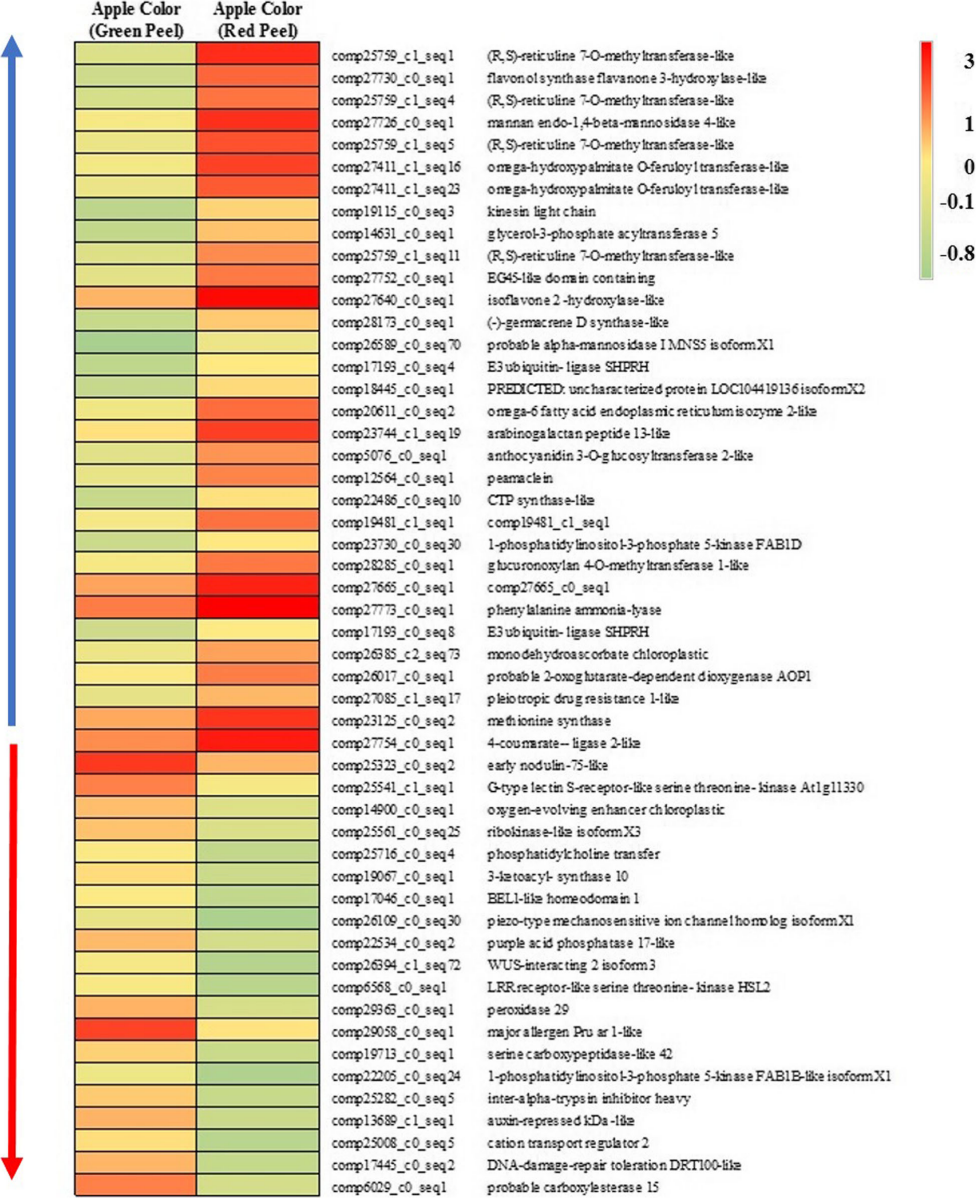
Heatmap of log transformed FPKM values of differentially expressed transcripts in red peel compared to green peel in Apple Color cultivar. Blue arrow represents up-regulated and red represents downregulated transcripts in red peel

Apple color skin of AC guava develops within a short time period of ~ 3–5 days in winter season during fruit maturation. Comparison of FPKM value of transcripts belonging to red vs green skin, identified only 52 DETs indicating very specific pathways involved in fruit color development (Fig. 6; Additional file 13: Data S6). Interestingly, all the transcripts of phenylpropanoid and lignin pathway showed up-regulation indicating the color development in the skin of guava is result of over expression of phenypropanoid and lignin biosynthesis pathway (Additional file 10: Figure S5; Additional file 13: Data S6). Comparison of PP mature fruit with AS mature fruit identified 19 DETs with 9 up-regulated and 10 down-regulated transcripts (Additional file 13: Data S7). However, only omega-hydroxypalmitate O-feruloyl transferase-like indicated a footprint of secondary metabolism pathway.

### Fruit color development in colored guava genotypes, is concomitant with ripening process

We hypothesized that pink pulp / apple color skin development may share the fruit ripening related genes as the color development in colored cultivars is concomitant with ripening process. The cluster analysis of DETs in 5 comparisons viz. AS ImF vs 0DF, 3DF vs 0DF, 7DF vs 3DF, PP ImF vs 0DF, and AC_RP vs AC_GP was carried out. AS mature fruit, PP mature fruit, AC green peel and AC red peel clustered in single group of mature fruit stages. AS 3DF and AS 7DF made another group with mostly similar expressions except 11 DETs. Third cluster consisted of AS ImF and PP ImF, again a result of similar fruit stage (Additional file 11: Figure S6; Additional file 13: Data S8).

**Fig. 7:**
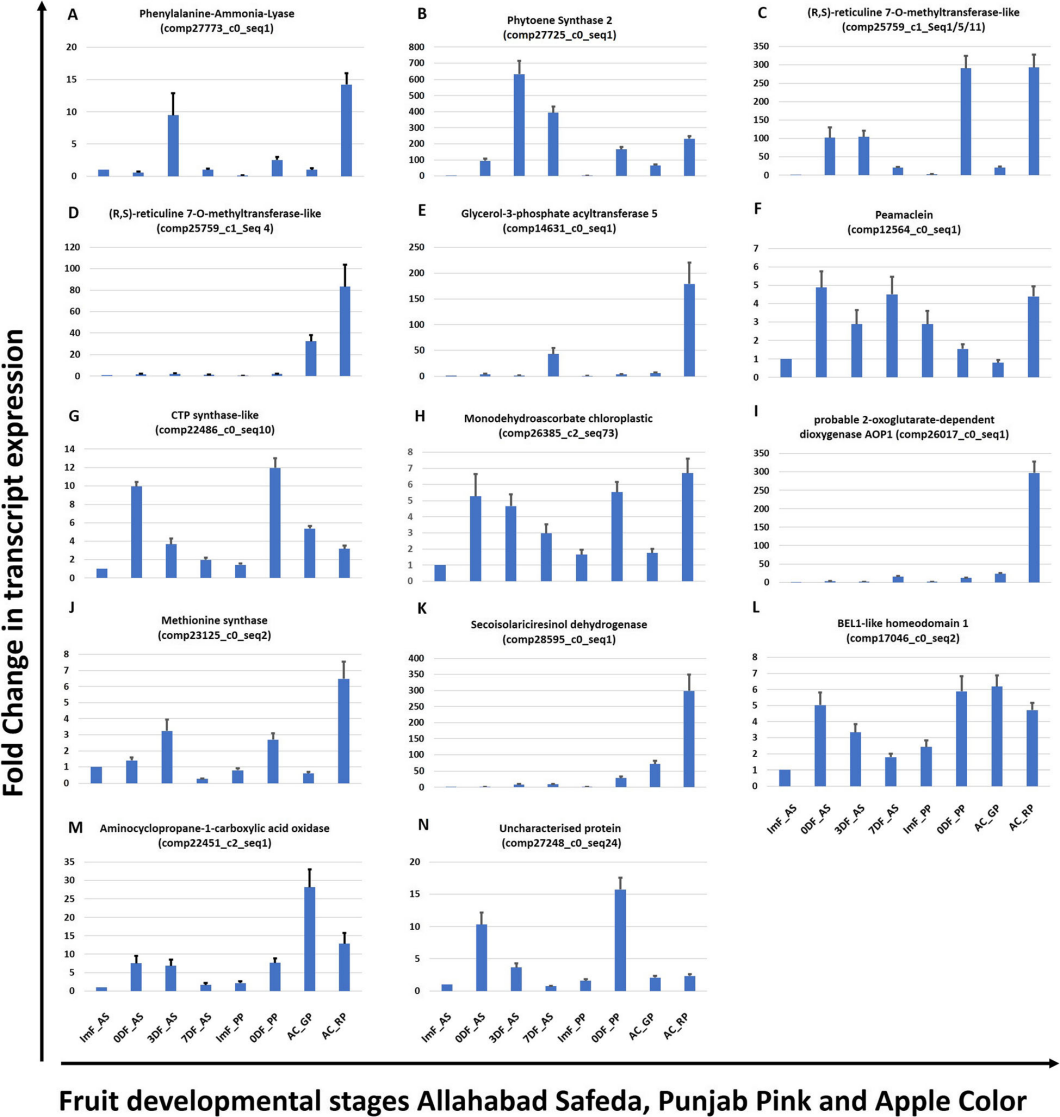
qRT-PCR assay of universal fruit color determining factors in cereals, fruits and vegetables **a** Phenylalanine Ammonia-Lyase, **b** Phytoene Synthase 2 and candidate genes for color development in apple color skin of guava cv. Apple Color and pink pulp in Punjab Pink **c** & **d** (R,S)-reticuline 7-O-methyltransferase-like, **e** Glycerol-3-phosphate acyltransferase 5, **f** Peamaclien **g** CTP synthase-like, **h** Monodehydroascorbate chloroplastic, **i** Probable 2- oxoglutarate-dependent dioxygenase AOP1, **j** Methionine synthase, **k** Secoisolariciresinol dehydrogenase, **l** BEL1-like homeodomain 1, **m** Aminocyclopropane-1-carboxylate oxidase 1-like, **n** Uncharacterized protein LOC104449412 (transcript id are given in brackets). Histone 3 is used as an internal reference. The fold change data is normalized to Allahabad Safeda immature fruit set to unity. Tissue types compared are: ImF_AS – Immature fruit of Allahabad Safeda, 0DF_AS – Mature fruit of Allahabad Safeda at harvesting stage, 3DF_AS – Fruit 3 days after harvesting, 7DF_AS – Fruit 7 days after harvesting, ImF_PP – Immature fruit of Punjab Pink, 0DF_PP – Mature fruit of Punjab Pink at harvesting stage, AC_GP – Green peel of Apple Color fruit 5 days before harvesting stage of fruit, AC_RP – Red peel of Apple Color fruit at harvesting stage. Error bars represents ±Standard error with *n* = 3

qRT-PCR for two well-known candidates for coloration in fruits and vegetables PAL and PSY 2 was carried out to see the reproducibility of differential FPKM expression values. Expression of PAL in AS fruit was maximum at yellow peel color ripe stage (~ 9.5X in 3DF_AS compared to ImF_AS; Fig. 7a). However, expression in mature Punjab Pink fruit (0DF_ PP with pink pulp) was ~ 1.8 fold higher as compared to mature Allahabad Safeda fruit (0DF_AS). Interestingly, expression in red peel of Apple Color (AC_RP) was found the highest (~ 1.5 X compared to 3DF_AS). These results indicated that the expression of PAL increases with ripening, but is also genotype dependent and might have contribution towards red coloration in peel of Apple Color genotype. However, Phytoene Synthase 2 (Fig. 7b) shows a general trend of increase in expression with maturity and ripening in all the three genotypes. These results also indicated that observations recorded in our comparative transcriptomic data are in line with qRT-PCR analysis.

### Candidate genes for color development in guava

To identify the genes involved in color development, GO enrichment of DETs was performed (Additional file 12: Figure S7). For the potential candidates FPKM values were compared in red peel of Apple Color AC_RP vs green peel AC_GP and mature fruit of Punjab Pink 0DF PP vs mature fruit of Allahabad Safeda 0DF AS for all the tissue types. The FPKM values showed higher expression of genes at color turning stages (Additional file 4: Table S4). Interestingly, the FPKM comparative analysis indicated reticulin o-methyltransferase (responsible for converting alkaloid reticulin to laudanine) as top candidate gene. The moderate expression of transcripts was present in green and maturing fruits of AS. However, huge increase in expression of reticulin o-methyltransferase in red peel of AC and mature PP fruit suggested increased levels of such alkaloids in colored fruit tissues. Maximum FPKM expression value of glycerol-3-phosphate acyltransferase 5 (GPAT 5), peamaclein, CTP synthase-like, chloroplastic monodehydroascorbate (MDA), probable 2-oxoglutarate-dependent dioxygenase AOP1 (2OG-AOP1) and methionine synthase (MS) corresponding transcripts in red peel of guava suggested interplay of several candidate genes for red coloration in peel of guava. Higher expression of transcripts for Secoisolariciresinol dehydrogenase (SDH), BEL1-like homeodomain 1(BLH1) in PP indicates additional candidates for red color in pulp of guava.

Three transcripts (R, S)-reticuline 7-O-methyltransferase-like (comp25759_c1_Seq1, comp25759_c1_Seq5 & comp25759_c1_Seq11) showed high homology, so common primers were designed for qRT-PCR. Results show that expression of (R, S)-reticuline 7-O-methyltransferase Like (RML) transcripts increases to 100X at 0DF_AS compared to ImF_AS set to unity (Fig. 7c). However, expression did not change in 3DF_AS compared to 0DF_AS and reduced to 20X in 7DF_AS. Interestingly, expression in ImF_PP is ~3X compared to ImF_AS and increases to ~ 300 X in 0DF_PP. Expression in AC_GP is ~21X but show an increase to ~300X in AC_RP, almost to similar levels as in 0DF_PP suggesting RML, a candidate for coloration in both guava peel and flesh. Surprisingly, expression of RML_ comp25759_c1_seq4 (Fig. 7d) stayed at low level in AS and PP at all the stages, however, its expression is ~30X in AC_GP and reaches to 80X in AC_RP. In AC this result indicated genotype specificity of RML members. High expression of RML family members emphasized their role in color development in guava. High expression of G3PAT, SDH, AOP1, MS and SDH in AC_RP indicated the additional candidates for color development in guava (Fig. 7). In general, qRT-PCR for all these candidates supported our transcriptome based FPKM results.

## Discussion

### Fruit ripening and maturation involves ABA, ethylene and secondary metabolite up-regulation

**Fig. 8:**
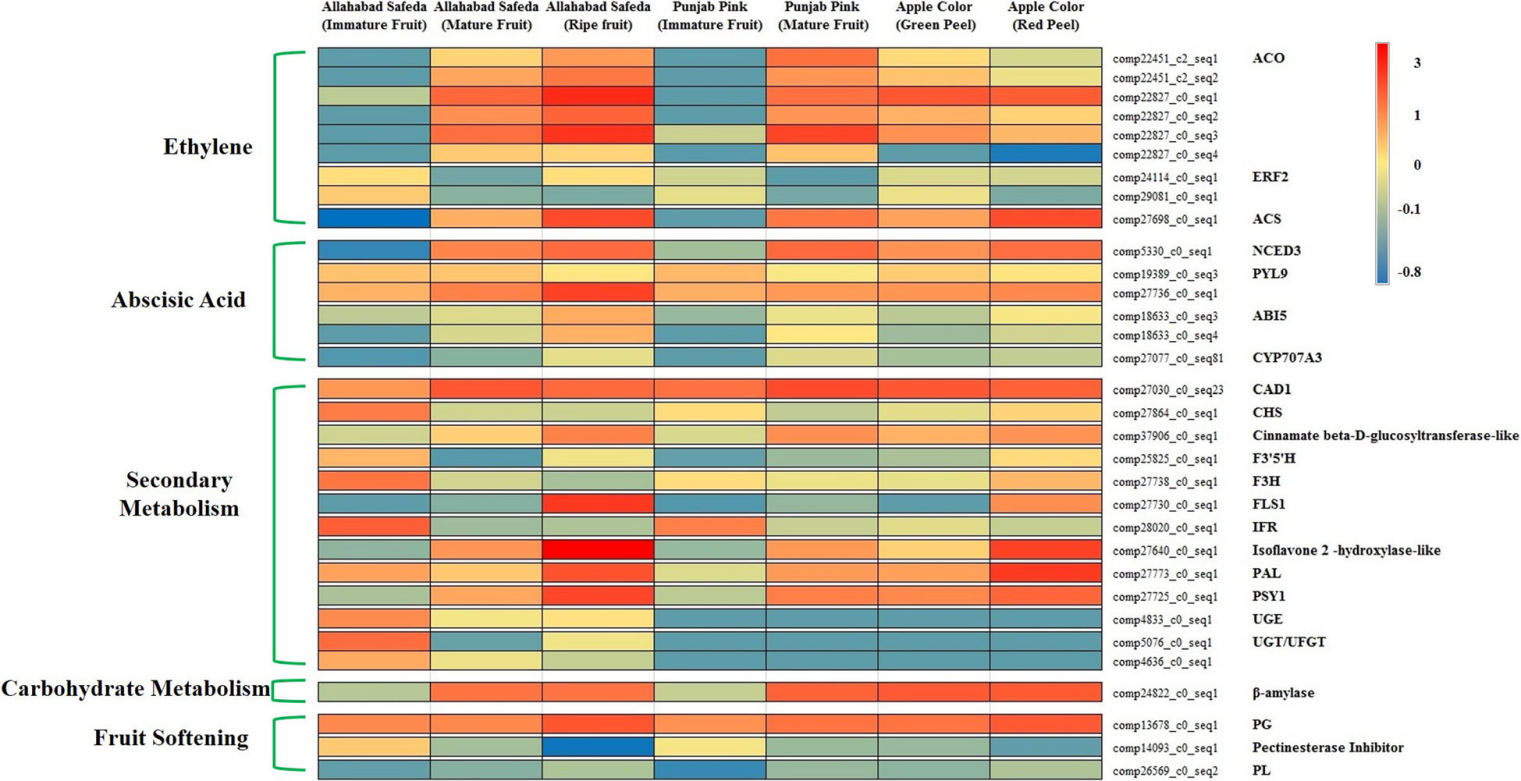
DETs mapped to fruit maturation and ripening in Allahabad Safeda, Punjab Pink and Apple Color. Ethylene biosynthesis and signaling (ACO, 1-aminocyclopropane-1-carboxylate oxidase; ERF2, ethylene-responsive transcription factor 2; ACS, 1-aminocyclopropane-1-carboxylate synthase), Abscisic acid biosynthesis and signaling (NCED3, 9-cis-epoxycarotenoid dioxygenase 5; PYL9, abscisic acid receptor PYL9; ABI5, Abscisic acid-insensitive 5 7; CYP707A3, abscisic acid 8-hydroxylase 3), secondary metabolism (CAD1, probable cinnamyl alcohol dehydrogenase 1; CHS, chalcone synthase; Cinnamate beta-D-glucosyltransferase-like; F3’5’H, flavonoid 3,5 -hydroxylase; F3H, flavanone 3-hydroxylase; FLS1, flavonol synthase flavanone 3-hydroxylase; IFR, isoflavone reductase; Isoflavone 2 -hydroxylase-like; PAL, phenylalanine ammonia-lyase; PSY1, phytoene synthase chloroplastic-like; UGE, UDP-glucose 4-epimerase; UGT/UFGT, anthocyanidin 3-O-glucosyltransferase), carbohydrate metabolism (β-amylase) and fruit softening (PG, polygalacturonase; Pectinesterase Inhibitor,; PL, pectate lyase 4). The color scale on the right represents the log-transformed FPKM values

Climacteric fruits show increased rate of respiration and ethylene biosynthesis which in turn triggers the activity of enzymes like PGs, PeLs and, pectate methylesterases (Pme). Process of ripening is accelerated by the conversion of complex polysaccharides into simple sugars leading to increased sugar to acid ratio concomitant with textural and color changes. Conversion of l-aminocyclopropane-l-carboxylic (ACC) acid from S-adenosylmethionine (SAM) is catalyzed by ACC synthase and is the rate limiting step in ethylene biosynthesis [44, 45]. Expression value comparison in AS, PP and AC showed differential increase in transcripts corresponding to ACO 1, ACO 2, ACS and ETR2 during fruit maturation (Fig. 8; Additional file 13: Data S9). ABA biosynthesis and signaling is also found up-regulated during fruit maturation and ripening [46]. We observe increased expression of ABA biosynthesis gene NCED, receptor Pyrabactin resistance 1 like 9 (PYL9) and TF ABA insensitive 5 (ABI5) during fruit ripening in all 3 cultivars (Fig. 8; Additional file 13: Data S9). Ethylene and ABA are stress hormones and up-regulates defense system of plants against pathogens by stimulating phenylpropanoid pathway, pathogenesis-related proteins and inducing systemic resistance [47]. Expression of pathogenesis related PR-4 is high in fruit tissues with the highest expression in red peel and PR-10 in green peel (Additional file 13: Data S9).

Guava is rich in secondary metabolites. Expression of genes controlling secondary metabolites like isoflavone reductase (IFR) is maximum in immature fruits, flavanone 3-hydroxylase (F3H) is high in leaf, flower and immature fruits and flavonoid 3, 5 -hydroxylase (F3’5’H) 2-like in leaf and flower tissue (Additional file 13: Data S9). Interestingly, expression of PAL, isoflavone 2 -hydroxylase-like and flavonol synthase are high during fruit maturation with maximum expression in ripe guava (Fig. 8; Additional file 13: Data S9). Hydrolysis of starch by β-amylase generates maltose leading to sweet flavor in ripened fruits [8]. Expression of β-amylase increased at 0DF and 3DF while reduced at over-ripe stage 7DF. Also, the expression is much higher in AC peel and pink PP fruit (Fig. 8; Additional file 13: Data S9). High expression of β-amylase underscores the sweet-smelling nature of guava at fruit ripening in general and specifically higher in AC and PP cultivars compared to AS.

**Fig. 9:**
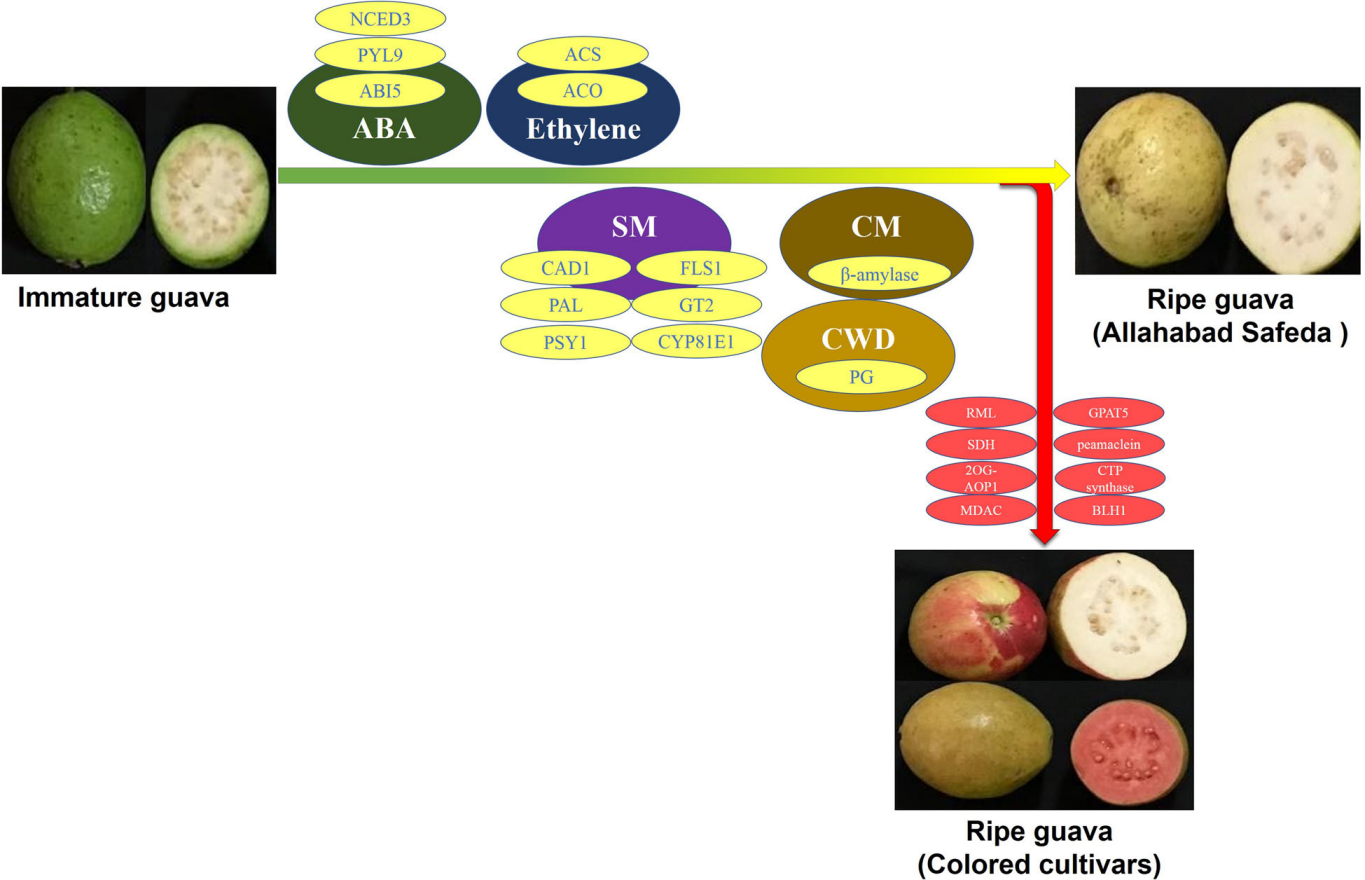
Concordant model for ripening of guava in Allahabad Safeda and color development in colored cultivars Punjab Pink and Apple Color based on RNA-Seq (FPKM) and/or qRT-PCR results. Abscisic Acid (ABA) biosynthesis and signaling (NCED3, 9-cis-epoxycarotenoid dioxygenase 3; PYL9, Pyrabactin like 9; ABI5, Abscisic acid-insensitive 5)**,** Ethylene biosynthesis (ACS, 1-aminocyclopropane-1-carboxylate synthase; ACO, 1-aminocyclopropane-1-carboxylate oxidase), secondary metabolism (SM) (CAD1, cinnamyl alcohol dehydrogenase 1; PAL, phenylalanine ammonia-lyase; PSY1, phytoene synthase 1; FLS1, flavonol synthase 1; GT2, cinnamate beta-D-glucosyltransferase, CYP81E1: Isoflavone 2 -hydroxylase), carbohydrate metabolism (CM) (β-amylase) and cell wall degradation (CWD) (PG, polygalacturonase) pathways interact for fruit ripening in guava. ABA, Ethylene, SM, CM and CWD further interact with (R,S)-reticuline 7-O-methyltransferase (RML), glycerol-3-phosphate acyltransferase 5 (GPAT5), Peamaclein, CTP synthase, Monodehydroascorbate chloroplastic (MDAC), 2- oxoglutarate-dependent dioxygenase (2OG) AOP1, Secoisolariciresinol dehydrogenase (SDH) and BEL1-like homeodomain 1 (BEH1) for color developement in colored genotypes

Synthesis of lignin monomers involve the phenylpropanoid pathway initiated by PAL and followed by Cinnamate 4-hydroxylase (C4H), 4CL, HCT, Caffeoyl CoA 3-O-methyltransferase (CCoAOMT), Cinnamoyl CoA reductase (CCR), Caffeic acid 3-O-methyltransferase (COMT) and CAD genes. Several CAD family members generally show up-regulation in the fruit flesh and respond to ABA, ethylene and various biotic and abiotic stresses as observed in melon [33]. However, expression of COMT gene is directly associated with increase in lignin content and is maximum in immature AS fruit. HCT and CAD genes control the crucial step in suberin [30, 31] and cutin biosynthesis [32]. HCT and CAD genes are the most up-regulated transcripts (Additional file 3: Table S3) in MFr vs LSt. Their expression increases during maturation and was the highest at ripe fruit stage followed by reduction at over-ripe stage indicating lignin, suberin and cutin synthesis during maturation and ripening. PGs expression increases during ripening. We also identified the highest expression of transcript corresponding to PGs in overripe fruit of AS, and high expression in red peel of AC compared to green peel and mature compared to immature fruit in PP (Fig. 8; Additional file 13: Data S9). Results of comparative transcriptome analysis in this manuscript are in concordance with protein and metabolite analysis reported in different fruit species supporting that this first transcriptome of guava will play a promising role in setting the stage for functional genomics in guava. Our findings of gene up-regulation during ripening of guava from immature fruits are in concordance with existing literature and are summarized in Fig. 9. Maturation of guava involves the wave of ABA biosynthesis and signaling followed by ethylene biosynthesis and accompanies the secondary metabolites accumulation, upregulation of carbohydrate metabolism and cell wall degradation enzymes.

### (R,S)-reticuline 7-O-methyltransferases are the potential candidates for color development in guava

Transcript FPKM value and qRT-PCR (Fig. 7; Additional file 4: Table S4) of RML, GPAT 5, Peamaclein, CTP synthase-like, chloroplastic MDA, 2OG-AOP1, MS and SDH genes show that expression level of these are maximum in AC fruit skin begging the question if increased expression of these genes is responsible for colored guava skin. RMLs are involved in tetra hydrobenzyl isoquinoline alkaloid production in poppy at the step of reticuline to laudenine conversion [48]. Up-regulation of 4 isomeric transcripts of RML in peel of guava suggests that red guava fruits may serve as a good source for extracting alkaloids like reticulin and laudenin rather than a much-regulated source like poppy. Although the reticulin and laudenin are extracted from poppy for medicinal purposes as an anti- diarrheal, anti-dysenteric, anticough, the use of guava fruit for similar utility have been described before [10, 13]. BLAST search of four RML transcripts identified its homologs in eucalyptus, grape and cork oak tree with 74–86% identity. Expression of all the four RML transcripts is the second highest in PP 0DF- mature fruit (pink pulp) after AC_RP - red peel (Additional file 4: Table S4).

GPAT 5 is required for synthesis of suberin in Arabidopsis root and seed coat [49]. Increased expression of HCT and GPAT 5 indicates escalated suberisation occurring in fruit coat in a short duration accompanying the ripening process. Peamaclein originally identified in peach fruit, is a recently identified gibberellin induced fruit allergen conserved among various fruits [50]. Higher expression of peamaclein transcripts in red peel (Additional file 4: Table S4) might be an indicator of high fruit allergen prevalence in red peel. CTP synthase-like is an enzyme associated with cytosine synthesis. Increased CTP cellular level in yeast has been associated with increased phospholipid synthesis via the Kennedy pathway [51]. This suggests increased levels of phospholipids in apple color fruit skin.

MDA can be reduced to ascorbate through reactions by MDA reductase (MDAR) in chloroplast and help relieving the oxidative stress by reactive oxygen species (ROS) evolving from the photosynthesis reactions [52]. Overexpression of MDAR in tomato has been associated with stress enhanced tolerance to temperature and methyl viologen-mediated oxidative stresses [52]. High expression of chloroplastic MDA transcript indicates the release of ROS in the fruit peel during color change. 2OG-AOP1 is involved in glucosinolate synthesis in Arabidopsis. Interestingly, 2OG is also the second largest enzyme family in plants involved in hormone and flavonoid biosynthesis [53]. Increased expression of 2OG-AOP1 is convincingly in the line of increased expression of flavonoid biosynthesis related genes during color turning in AC.

Methionine not only acts as a building block for protein synthesis but also serves as immediate precursor of S-adenosylmethionine, a major methyl-group donor in transmethylation reactions and as an intermediate for biosynthesis of polyamines and ethylene [54]. Up-regulation of methionine synthase, an enzyme catalyzing the last step of methionine biosynthesis supports the increased demand of ethylene biosynthesis during fruit ripening in general and highest expression in red skin (Fig. 7j; Additional file 4: Table S4).

SDH converts (2)-secoisolariciresinol into (2)-matairesinol in *Forsythia intermedia*, a precursor for the biosynthesis of antiviral and anticancer agent, podophyllotoxin [55, 56]. Although, SDH is identified in a comparison of AS 0DF to PP 0DF but the expression was the highest in AC red skin with > 3-fold expression increase compared to green skin (Fig. 7k; Additional file 4: Table S4). Presence of RML and SDH high expression in red guavas indicated colored guava being a rich source of rare secondary metabolites. BLH1 have been implicated in leaf and ovule development in Arabidopsis [57] and overexpression of apple BEL1-Like MDH1 in Arabidopsis [58] leads to reduced fertility and other pleiotropic effects, however no implication in color development are reported. RNAi of *Sl*BEL1- like 11 in tomato increased the chlorophyll content [59] and delayed fruit ripening. Increased expression of BEL1 in PP pulp might have role in chromoplast development. Comp27248_c1_seq24 is another highly expressed transcript in PP 0DF that does not have any other functional evidence but shows 90% identity with *Eucalyptus grandis* uncharacterized LOC104449412.

### Up-regulation of DETs in red skin of guava *cv.* Apple color implicates cross talk among myriad dynamic processes

Up-regulation of Mannan endo-1,4-beta-mannosidase (β-mannanase) indicates utilization of mannans in producing mannose oligosaccharides from lignocellulosic polysaccharides [60]. Kinesis light chains (KLCs) are involved in microtubule mediated organelle transport in eukaryotes [61]. Up-regulation of KLC corresponding transcripts indicate high metabolic activity during skin color alteration in guava. EG45-like domain proteins belong to plant natriuretic peptides (PNPs), a class of systemically mobile molecules distantly related to expansins with implication in regulating water and ions homeostasis and participation in plant immune response [62], so are in line with increased demand in leathery red skin. (−)-germacrene D synthase is involved in biosynthesis of a sesquiterpene germacrene D generally obtained from cubebe oil [63]. Golgi α-mannosidase II is a key glycosyl hydrolase in the N-linked glycosylation pathway [64], indicating N-glycosylation role in reddening of skin. E3-ubiquitin ligases catalyze final step of ubiquitin conjugation demanding tight regulation to ensure accurate substrate ubiquitylation and finally degradation via 26 S proteasomal pathway marking an important checkpoint for protein turnover [65]. Up-regulation of 2 transcripts belonging to E3 ubiquitin- ligase SHPRH in apple color skin development indicated high turnover of specific proteins. Omega-6 fatty acid destaurases or Fatty Acid Destuarse 2 (FAD2) desaturates oleic acid to linoleic acid (18:2) for enhancing stress tolerance in endopasmic reticulum in response to abiotic stresses [66]. Omega-6 fatty acid endoplasmic reticulum isozyme 2-like up-regulation in red and leathery skin might be an indicator of better protection to biotic and abiotic stresses. Arabinogalactan peptides (AGPs) in wheat are encoded by grain softness protein genes [67]. Wheat-AGPs derived down-regulation of GSPs in RNAi lines increases the grain hardness and decreases viscosity of aqueous extracts. Such extension like activities in the reddening skin might be attributes of AGPs as Arabinogalactan peptide 13-like is up-regulated. Knock-down of FAB1A/B, 1-phosphatidylinositol-3-phosphate 5-kinase in Arabidopsis causes defect in the membrane recycling by auxin transporters [68]. FAB1 also plays an important role in endosomes maturation for mediating cortical microtubule association of endosomes [69]. Increased expression of 1-phosphatidylinositol-3-phosphate 5-kinase/FAB1D during color change is suggestive of increased auxin transport. Hemicellulose 4-O-methyl glucuronoxylan is a major component present in the secondary cell walls of eudicots. Arabidopsis GXMT catalyzes 4-O-methylation of the glucuronic acid substituents in hemicellulose [34]. Up-regulation of GXMT 1-like indicates hemicellulose synthesis at color change stage.

### Phenylpropanoid pathway in colored guava

Anthocyanin biosynthesis genes like F3H [70], Isoflavone-2′-hydroxylase [71], Anthocyanidin 3-O-glucosyltransferase 2-like / UGT [72], 4-Coumarate:CoA ligase and PAL are up-regulated in red skin compared to green skin (Fig. 6; Fig. 8; Additional file 13: Data S6). However, expression of multiple transcripts corresponding to these genes are also up-regulated during fruit ripening in AS and PP, pointing the shared phenomenon between fruit ripening and color accumulation. However, doubled expression of PAL in AC red skin compared to AS 3DF (Fig. 7a; Additional file 13: Data S9) compels the idea that increased anthocyanin accumulation in red skin might be the result of PAL accumulation. PAL is the check point between primary and secondary metabolism and activity increases in response to biotic and abiotic stresses. PAL in combination with other phenylpropanoid enzymes like 4-Coumarate-CoA ligase, Chalcone synthase, Cinnamic acid 4-hydroxylase, F3H, Flavonol synthase, Stilbene synthase, Isofavone synthase, Resveratrol synthase etc. has been used in different hosts, like *Escherichia coli*, *Saccharomyces cerevisiae*, *Pseudomonas putida* and *Streptomyces spp.* to synthesize a wide range of phenylpropanoid-derived compounds like flavanones, naringenins, kaempferol, quercetin, stilbenes and many more [73]. Preliminary work of oral or subcutaneous administration of PAL to Phenyl Ketone Uric patients leading to substantial reduction of plasma L-Phe levels has been reported [73]. Increased expression of PAL in red peel indicates a potential new source of this enzyme in guava. Expression increase in PSY and ACO (Fig. 7; Additional file 13: Data S9;) during ripening in AS, PP and AC supports high correlation in color development and maturation in guava but might need interaction with specific factors like RML / GPAT 5 / peamaclein / CTP synthase / MDA chloroplastic / 2OG-AOP1 / SDH / BLH1 (Fig. 9).

## Conclusions

Tissue specific and genotypic comparative transcriptome analysis of guava reported here corresponds to the metabolic changes expected and observed in climacteric fruit crops. The transcriptome assembly generated in this study will set the stage for functional genomics in guava, the second most important fruit crop of Northern India. Comparative transcriptome sequence analysis of non-colored vs colored guava cultivars will lead to identification of simple sequence repeat, Insertion-deletion and single nucleotide polymorphism-based markers and be utilized in linkage mapping and other genetic studies. Notably, identification of candidate gene-based markers for red color will aid in generating polymorphic markers and prove a boon for marker assisted breeding for color trait in guava.

## Methods

### Original source of the plant materials

Punjab Pink is a hybrid between Portugal x L-49 = F1 x Apple Color and was developed by Punjab Agricultural University, Ludhiana and released in year 2009. CISH-G5/Lalima is an apple color selection of Central Institute of Sub-tropical Horticulture, Lucknow, Uttar Pradesh, India. Allahabad Safeda is an open pollinated seedling selection from Uttar Pradesh, India. The three cultivars were clonally propagated and raised in the mother block of Regional Fruit Research Station - PAU, Bahadurgarh, Patiala, Punjab, India. The 10 years old clonal propagated trees growing in fruit research orchards of PAU, Ludhiana, India have been used in current study.

### Source of fruits or plant material and tissue sample collection

In the present study we used *Psidium guajava* L. local cultivars Allahabad Safeda (AS), Apple Color (AC: CISH-G5) and Punjab Pink (PP) 10 years old trees growing in Orchards of PAU, Ludhiana, India. Allahabad Safeda is the most prominent cultivar grown throughout India for table purpose. AS fruits are green skinned at immature and harvesting stage (mature fruit, 0DF), starts turning yellow and skin turns completely yellow within 3 days after picking (ripe fruit, 3D) and becomes over-ripe thereafter. Foliage and flower buds of AS, PP and AC are all green however pulp of PP turns to deep pink on maturity (0DF), whereas immature fruit pulp is white. Fruits of AC turn their skin color from green (−5DF) to reddish (0DF) in winter season (Fig. 1).

In AS, we collected actively growing immature young leaves, fully expanded mature leaves, actively growing shoot tips, flower buds at six different developmental stages from immature to mature (1 day before anthesis) and 0D opened flower, 80DPA (immature fruit) with seeds and without seed (ImF), fully mature ready to harvest fruit tissue with seeds and without seeds (0DF), fruit tissue 3 days after harvesting without seeds (3DF) and fruit tissue 7 days after harvesting without seeds (7DF) in three replicates. All the tissues were flash frozen in liquid N_2_ and stored in − 80 °C before proceeding to RNA extraction. PP immature fruit (80 DPA) without seed (ImF_PP), mature fruit without seed (0DF_PP) and AC green peel (AC_GP, −5D fruit) and red peel (AC_RP, 0D fruit) were also harvested, similarly.

### RNA extraction

Total RNA was extracted from all the tissues using Spectrum™ Plant Total RNA Kit (Sigma-Aldrich) followed by on-column DNase I digestion (Sigma-Aldrich) for removing DNA contamination. RNA integrity was analysed on 1.2% agarose denaturing gel as described before [74]. High quality RNA from different tissues was pooled in equimolar ratios to reduce the number of libraries to be sequenced. Briefly, total RNA extracted independently from immature young leaves, fully expanded mature leaves and actively growing shoot tips was pooled in equal amount to make one RNA sample Leaf shoot tip (LSt), 6 flower bud stages and opened flower sample- Mixed flower bud (MFb), 80 DPA immature fruit (ImF), 0D fruit (0DF), 3D ripe fruit (3DF) and 7D overripe fruit (7DF) sample- Mixed fruit (MFr). All the fruit stage samples used to pool MFr were a slice of guava fruit containing peel, pulp and seed altogether. However, independent fruit samples ImF, 0DF, 3DF and, 7DF were also sampled with skin but without seed. So, in total 7 samples representing 7 tissue types of AS were LSt, MFb, MFr, ImF, 0DF, 3DF and 7DF. For PP and AC we extracted 2 RNA samples each ImF_PP & 0DF_PP and AC_GP & AC_RP, respectively.

### Library preparation, RNA sequencing and reference assembly generation

Bioanalyzer (Agilent) was used to asses RNA quality before preparing RNA-Seq libraries. Libraries were constructed with ribo-zero treated RNA from 3 biological replicates of AS LSt, MFb, MFr and a single sample for AS ImF, 0DF, 3DF, 7DF, ImF_PP, 0DF_PP, AC_GP & AC_RP. RNA had RIN value ≥8.3 and TruSeq Stranded RNA Library Prep kit (Illumina) was used with an insert size of ~ 300 bp. The paired end (PE) libraries were sequenced on Illumina HiSeq2500. 100 bp high quality PE reads were generated. All the libraries were run in a single lane to avoid any discrepancies while calculating differential expression (FPKM). Read quality was assessed using FASTQC toolkit [75, 76]. Adapter and low quality sequences were trimmed at minimum PHRED quality score 30 using Trimmomatic read filtering tool [77]. de novo RNA-seq assembly was generated by pooling AS libraries of LSt, MFb, MFr, ImF, 0DF, 3DF, 7DF with Trinity transcriptome assembler [27] as no reference assembly for guava is available. Benchmarking Universal Single-Copy Orthologs (BUSCO) analysis was performed using BUSCO pipeline with eudicot model to estimate the completeness of transcriptome assembly.

### Functional annotation of de novo assembled Allahabad Safeda Transcriptome

The assembled transcripts were annotated on the basis of corresponding homologs identified from BLASTX [78] program with search against NCBI protein “nr” database at e-value of 1e^− 3^. Gene ontology (GO) terms associated with transcripts were determined using BLAST2GO program [79]. GO enrichment was done among the DETs in specific comparisons using GOseq [80]. KEGG annotations were also assigned using Blast2GO KEGG mapping. Transdecoder tool in Trinity package was used to identify longest open reading frame (ORF) and protein families were assigned by searching against the Pfam database using pfamscan [81]. Datafile with transcript ID description, GO annotation and enzyme name are provided in Additional file 13: Data S1.

### Measuring gene expression and identification of differentially expressed genes/transcripts

The trimmed reads were mapped to reference transcriptome and abundance of transcripts was measured in FPKM values using RSEM estimation tool, for each sample [82]. Trimmed mean of M-values (TMM) normalization for libraries was performed with Trinity. The expected count values were used for determining the differential expression with edgeR [83] on biological replicated data. Default dispersion value of 0.1 was used to calculate the differential expression for samples with no replicates. Transcripts exhibiting ≥4-fold change (Log2FC) in expression and < 0.001 false discovery rate (FDR) were considered significant DETs among different tissues, developmental stages and /or genotypes.

### Pathway analysis

DETs in tissues/genotypes were put together in a fasta file and pathway annotation were determined using online Mercator analysis tools (http://mapman.gabipd.org) [84]. DETs were binned into functional categories by Mercator. MAPMAN software [43] was used for graphical representation of metabolic and signaling pathways. The results of metablolic pathway analysis described in manuscript can be reproduced by installing MAPMAN and uploading the data set in supplementary files on a computer following authors guidelines described in original manuscript [43].

### qRT-PCR analysis

Two micro grams of RNA was reverse transcribed by reverse transcriptase (Maxima First Strand cDNA Synthesis Kit for RT-qPCR containing oligo (dT)18 and random hexamer primers; Thermo-Scientific, Surrey, UK), in a 25 μl reaction further diluted to 100 μl with nuclease free water. Diluted cDNA template (2 μL) was used for a 15 μl PCR reaction. Gene-specific primers (Additional file 5: Table S5) for candidate genes were used to amplify cDNAs. Histone 3 (comp27670_c0_seq1) was used as internal control. qRT-PCR was performed using PowerUp™ SYBR™ Green Master Mix on a LightCycler® 480 Instrument. Fold change was calculated as described before [85]. Oligonucleotide primers (Additional file 5: Table S5) were designed using Primer3 design (http://frodo.wi.mit.edu/).

## Supplementary information

**Additional file 1: Table S1.** Description of RNA-Seq paired-end data through Illumina high-throughput sequencing.

**Additional file 2: Table S2.** Correlation matrix values among Leaf Shoot tip (LSt), Mixed Flower bud (MFb) and Mixed Fruit (MFr) tissue samples and the replicates.

**Additional file 3: Table S3.** Expression of top 20 co-up regulated transcripts in Allahabad Safeda fruit tissue compared to leaf and flower with FDR < 0.001.

**Additional file 4:Table S4.** FPKM value of top differentially regulated transcripts of red peel vs green peel of Apple Color (AC) and Punjab Pink (PP) mature red fruit vs Allahabad Safeda (AS) mature fruit in development stages of AS, PP and AC.

**Additional file 5: Table S5.** Primer Sequences for candidate genes/internal control for qRT-PCR analysis.

**Additional file 6: Figure S1.** Summary of conserved orthologous genes (BUSCO) in the assembled guava transcriptome.

**Additional file 7: Figure S2.** Blast2GO distribution of assembled transcripts into A) Biological processes B) Cellular component C) Molecular function.

**Additional file 8: Figure S3.** Metabolic overview with MAPMAN analysis of differentially expressed transcripts of mature fruit (0DF) vs Immature fruit (ImF) of cv. Allahabad Safeda. Up- and Down- regulated DETs are represented with blue and red squares, respectively with log2 transformed values.

**Additional file 9: Figure S4.** MAPMAN analysis of differentially expressed transcripts of ripe fruit (3DF) vs mature fruit (0DF) of cv. Allahabad Safeda A) Metabolic Overview B) part of regulation overview C) proteasome and autophagy. Up- and Down- regulated DETs are represented with blue and red squares, respectively with log2 transformed values.

**Additional file 10: Figure S5.** MAPMAN analysis of differentially expressed transcripts of apple color skin vs green skin of Apple Color – CISH-G5, shows up-regulation of the secondary metabolism pathway. Up- and Down- regulated DETs are represented with blue and red squares, respectively with log2 transformed values.

**Additional file 11 : Figure S6.** Cluster analysis of differentially expressed transcripts among fruit stages of Allahabad Safeda (AS), Apple Color (AC) and Punjab Pink (PP) immature fruit (ImF), mature fruit (MF), 3 days after harvesting (3DF), 7 days after harvesting (7DF), green peel and red peel.

**Additional file 12: Figure S7.** Gene Ontology enrichments between A) red and green peel of Apple Color B) mature fruit of Punjab Pink and Allahabad Safeda.

**Additional file 13. **Guava_Reference_Transcriptome_Data files. **Data S1.** Functional Annotation of Allahabad Safeda Transcriptome Assembly. **Data S2.** Differential expression among different tissue types, mixed flower buds vs leaf & shoot tip (MFb vs LSt), mixed fruit stages vs leaf & shoot tip (MFr vs LSt) and mixed fruit stages vs mixed flower buds (MFr vs MFb) of Allahabad Safeda. **Data S3.** Differential expression between mature but unripened fruit (0DF) vs immature fruit (ImF) of Allahabad Safeda. **Data S4.** Differential expression between 3 days ripe fruit (3DF) vs mature unripened fruit (0DF) of Allahabad Safeda. **Data S5.** Differential expression between 7 days over-ripe fruit (7DF) vs 3 days ripe fruit (3DF) of Allahabad Safeda. **Data S6**. Differential expression between red peel vs green peel of Apple Color. **Data S7.** Differential expression between Punjab Pink mature fruit (PP_0DF) vs Allahabad Safeda mature fruit (AS_0DF). **Data S8.** FPKM values of DETs among fruit and peel stages of 3 genotypes. **Data S9.** Expression of genes involved in important pathways for fruit maturation, ripening and pathogenesis in different tissues of 3 guava genotypes.

## Data Availability

Trinity generated transcripts of length > 200 bp submitted to NCBI Transcriptome Shotgun Assembly (TSA) has accession no. GGPP00000000. RNA-seq data of *cv.* Allahabad Safeda is submitted under Bioproject PRJNA472130 with 13 biosamples (SAMN09227265–77). Raw reads are submitted under Short Reads Archive (SRA) database for LSt (SRR7186630, SRR7186631, SRR7186633), MFb (SRR7186632, SRR7186634, SRR7186635), MFr (SRR7186629, SRR7186636, SRR7186637), ImF (SRR7186628), 0DF (SRR7186640), 3DF (SRR7186639) and 7DF (SRR7186638). Reads for Punjab Pink (PP ImF - SRR7471728 & PP 0DF - SRR7471727) and Apple Color – CISH G5 (AC_GP - SRR7471740 & AC_RP - SRR7471739) can also be found at NCBI - SRA.

## Abbreviations

AC: Apple Color/CISH-G5/Lalima
AC_GP: Apple Color green peel
AC_RP: Apple Color red peel
AS: Allahabad Safeda
BUSCO: Benchmarking Universal Single-Copy Orthologs
DETs: Differentially expressed transcripts
FPKM: Fragments Per Kilobase Million
ImF: Immature fruit
ImF_PP: Immature fruit of Punjab Pink
LSt: Leaf and shoot tip
MFb: Mixed flower buds tissue
MFr: Mixed stage fruit tissue
PP: Punjab Pink
0DF_PP: Mature fruit of Punjab Pink
0DF: Mature ready to harvest fruit
3DF: 3 days after harvesting fruit
7DF: 7 days after harvesting fruit
