# Supplementary material for: RNA-sequencing based gene expression landscape of guava cv. Allahabad Safeda and comparative analysis to colored cultivars
Source: BMC Genomics. 2020 Jul 15;21:484. doi: 10.1186/s12864-020-06883-6 (PMC7364479; doi:10.1186/s12864-020-06883-6)
Supplement: Supplementary file 1 — Additional file 1: Table S1. Description of RNA-Seq paired-end data through Illumina high-throughput sequencing. [file 12864_2020_6883_MOESM1_ESM.docx]

**TABLE S1 Description of RNA-Seq paired-end data through Illumina high-throughput sequencing**

| **Genotype** | **Tissue type** | **Raw reads**  **(Millions)** | | | **Total Reads (Millions)** |
| --- | --- | --- | --- | --- | --- |
|  |  | **Replicate 1** | **Replicate 2** | **Replicate 3** |  |
| Allahabad Safeda | LSt | 11.92 | 13.93 | 11.57 | 137.3 |
|  | MFb | 9.05 | 8.47 | 9.60 |  |
|  | MFr | 8.95 | 10.88 | 8.86 |  |
|  | ImF | 12.0 |  |  |  |
|  | 0DF | 10.34 |  |  |  |
|  | 3DF | 10.23 |  |  |  |
|  | 7DF | 11.50 |  |  |  |
| Apple Colour | Green Peel | 10.35 |  |  | 20.24 |
|  | Red Peel | 9.89 |  |  |  |
| Punjab Pink | ImF | 9.66 |  |  | 20.0 |
|  | 0DF | 10.34 |  |  |  |
