# Supplementary material for: RNA-sequencing based gene expression landscape of guava cv. Allahabad Safeda and comparative analysis to colored cultivars
Source: BMC Genomics. 2020 Jul 15;21:484. doi: 10.1186/s12864-020-06883-6 (PMC7364479; doi:10.1186/s12864-020-06883-6)
Supplement: Supplementary file 2 — Additional file 2: Table S2. Correlation matrix values among Leaf Shoot tip (LSt), Mixed Flower bud (MFb) and Mixed Fruit (MFr) tissue samples and the replicates. [file 12864_2020_6883_MOESM2_ESM.docx]

**TABLE S2. Correlation matrix values among Leaf Shoot tip (LSt), Mixed Flower bud (MFb) and Mixed Fruit (MFr) tissue samples and the replicates**

|  | **LSt-R1** | **LSt-R2** | **LSt-R3** | **MFb-R1** | **MFb-R2** | **MFb-R3** | **MFr-R1** | **MFr-R2** | **MFr-R3** |
| --- | --- | --- | --- | --- | --- | --- | --- | --- | --- |
| **LSt-R1** | 1 | 0.967905 | 0.968402 | 0.407674 | 0.407639 | 0.423635 | 0.125528 | 0.130465 | 0.126424 |
| **LSt-R2** | 0.967905 | 1 | 0.969017 | 0.408846 | 0.40923 | 0.425321 | 0.133028 | 0.137092 | 0.133112 |
| **LSt-R3** | 0.968402 | 0.969017 | 1 | 0.407878 | 0.409019 | 0.423633 | 0.130904 | 0.133672 | 0.131271 |
| **MFb-R1** | 0.407674 | 0.408846 | 0.407878 | 1 | 0.936543 | 0.935906 | 0.194809 | 0.181779 | 0.181164 |
| **MFb-R2** | 0.407639 | 0.40923 | 0.409019 | 0.936543 | 1 | 0.945534 | 0.192801 | 0.183536 | 0.181287 |
| **MFb-R3** | 0.423635 | 0.425321 | 0.423633 | 0.935906 | 0.945534 | 1 | 0.182751 | 0.175526 | 0.173965 |
| **MFr-R1** | 0.125528 | 0.133028 | 0.130904 | 0.194809 | 0.192801 | 0.182751 | 1 | 0.980738 | 0.976851 |
| **MFr-R2** | 0.130465 | 0.137092 | 0.133672 | 0.181779 | 0.183536 | 0.175526 | 0.980738 | 1 | 0.978964 |
| **MFr-R3** | 0.126424 | 0.133112 | 0.131271 | 0.181164 | 0.181287 | 0.173965 | 0.976851 | 0.978964 | 1 |
