# Supplementary material for: RNA-sequencing based gene expression landscape of guava cv. Allahabad Safeda and comparative analysis to colored cultivars
Source: BMC Genomics. 2020 Jul 15;21:484. doi: 10.1186/s12864-020-06883-6 (PMC7364479; doi:10.1186/s12864-020-06883-6)
Supplement: Supplementary file 3 — Additional file 3: Table S3. Expression of top 20 co-up regulated transcripts in Allahabad Safeda fruit tissue compared to leaf and flower with FDR < 0.001. [file 12864_2020_6883_MOESM3_ESM.docx]

**TABLE S3 Expression of top 20 co-up regulated transcripts in Allahabad Safeda fruit tissue compared to leaf and flower with FDR <0.001**

| **Transcript ID** | **Log2Fold Change** | | **Functional Annotation** |
| --- | --- | --- | --- |
|  | **Fruit (MFr) vs Flower (MFb)** | **Fruit (MFr) vs leaf (LSt)** |  |
| comp27411_c1_seq16 | 15.29 | 13.80 | Hydroxycinnamoyl CoA shikimate / quinate hydroxycinnamoyltransferase-like protein |
| comp27411_c1_seq13 | 14.16 | 14.34 |  |
| comp27411_c1_seq30 | 13.56 | 13.75 |  |
| comp27411_c1_seq23 | 10.52 | 12.77 |  |
| comp27411_c1_seq8 | 12.10 | 12.29 |  |
| comp27411_c1_seq12 | 11.21 | 11.38 |  |
| comp22827_c0_seq3 | 9.6 | 14.99 | 1-aminocyclopropane-1-carboxylate oxidase |
| comp27730_c0_seq1 | 12.83 | 11.20 |  |
| comp27698_c0_seq1 | 12.16 | 12.52 |  |
| comp27085_c1_seq17 | - | 13.64 | ATP-binding cassette transporter |
| comp4924_c0_seq1 | 7.0 | 13.20 | Palmitoyltransferase PFA4 |
| comp28466_c0_seq1 | 7.2 | 12.69 | Subtilisin-like protease |
| comp27744_c0_seq1 | 8.5 | 12.60 | Expansin-like protein 45 |
| comp28304_c0_seq1 | 8.7 | 12.30 | Root cap protein 3 |
| comp19048_c1_seq4 | 9.6 | 12.30 | Cytokinin riboside 5_apos-monophosphate phosphoribohydrolase |
| comp27030_c0_seq2 | 4.8 | 12.28 | Cinnamyl alcohol dehydrogenase |
| comp5330_c0_seq1 | 10.14 | 11.60 | 9-cis-epoxycarotenoid dioxygenase 5 |
| comp24231_c0_seq2 | 8.0 | 11.57 | 2S albumin-like |
| comp27266_c1_seq13 | 8.3 | 11.55 | RbcX protein |
| comp27752_c0_seq1 | 2.7 | 11.53 | Blight-associated protein P12 (Fragment) |
| comp28285_c0_seq1 | 3.3 | 11.49 | Expressed protein |
