# Supplementary material for: RNA-sequencing based gene expression landscape of guava cv. Allahabad Safeda and comparative analysis to colored cultivars
Source: BMC Genomics. 2020 Jul 15;21:484. doi: 10.1186/s12864-020-06883-6 (PMC7364479; doi:10.1186/s12864-020-06883-6)
Supplement: Supplementary file 4 — Additional file 4:Table S4. FPKM value of top differentially regulated transcripts of red peel vs green peel of Apple Color (AC) and Punjab Pink (PP) mature red fruit vs Allahabad Safeda (AS) mature fruit in development stages of AS, PP and AC. [file 12864_2020_6883_MOESM4_ESM.docx]

**TABLE S4 FPKM value of top differentially regulated transcripts of red peel vs green peel of Apple Colour (AC) and Punjab Pink (PP) mature red fruit vs Allahabad Safeda (AS) mature fruit in development stages of AS, PP and AC**

| **Transcript ID** | **Description** | **Genotypes** | | | | | | | | | |
| --- | --- | --- | --- | --- | --- | --- | --- | --- | --- | --- | --- |
|  | | **Allahabad Safeda** | | | | | | **Punjab Pink** | | **Apple Colour** | |
|  |  | **LSt**  **Avg ^$^(S.E)** | **MFb**  **Avg ^$^(S.E)** | **ImF** | **0DF** | **3DF** | **7DF** | **ImF** | **0DF** | **GP** | **RP** |
| **AC peel Red vs Green Up-regulated** | | | | | | | | | | | |
| comp25759_c1_seq1 | (R,S)-reticuline 7-O-methyltransferase-like | 0.5 (0.3) | 0.3 (0.3) | 2.3 | 79.7 | 13.9 | 21.7 | 0 | 402.8 | 2.2 | 828.6 |
| comp25759_c1_seq4 |  | 0 | 10.5 (2.2) | 0 | 12.3 | 3.7 | 3.2 | 0 | 88.8 | 1.5 | 183.3 |
| comp25759_c1_seq5 |  | 0.2 (0.2) | 2.5 (0.5) | 0 | 66.1 | 16.5 | 24.6 | 0 | 220.4 | 5.2 | 382.1 |
| comp25759_c1_seq11 |  | 0.1 (0.1) | 0.4 (0.2) | 0 | 22.7 | 19.7 | 1.6 | 0 | 63.9 | 2.4 | 111.6 |
| comp14631_c0_seq1 | glycerol-3-phosphate acyltransferase 5 | 25.6 (0.8) | 1.2 (0.1) | 0.3 | 0.6 | 1 | 8.4 | 0.4 | 0 | 0.6 | 34.2 |
| comp12564_c0_seq1 | peamaclein | 71.8 (9.2) | 57.8 (0.8) | 2.6 | 18.3 | 34.5 | 46.9 | 1.8 | 18.4 | 4.3 | 130.3 |
| comp22486_c0_seq10 | CTP synthase-like | 0.4 (0.4) | 0 | 0 | 3.1 | 6.3 | 0 | 0 | 0 | 0.7 | 18 |
| comp26385_c2_seq73 | monodehydroascorbate chloroplastic | 6.4 (1.4) | 3.5 (2.8) | 3.2 | 18.9 | 1 | 26.9 | 1.2 | 28.6 | 5.5 | 69.4 |
| comp26017_c0_seq1 | probable 2-oxoglutarate-dependent dioxygenase AOP1 | 38.3 (1.6) | 97.4 (4.0) | 3.3 | 1.9 | 8.7 | 57.9 | 2.4 | 2.64 | 11.8 | 150.5 |
| comp23125_c0_seq2 | methionine synthase | 87.7 (3.8) | 168.3 (4.0) | 227.5 | 128 | 382.3 | 43.6 | 98.4 | 298.5 | 60 | 723.8 |
| **PP 0DF vs AS 0DF Up-regulated** | | | | | | | | | | | |
| comp28595_c0_seq1 | Secoisolariciresinol dehydrogenase | 67.8 (3.1) | 54.9 (2.1) | 6.2 | 1.3 | 21.4 | 42.1 | 1.3 | 66.8 | 302.9 | 1106.1 |
| comp17046_c0_seq2 | BEL1-like homeodomain 1 | 0.2 (0.2) | 1.4 (1.0) | 0 | 2.8 | 16.3 | 13.1 | 26.3 | 46.2 | 11.7 | 27.1 |
| comp22451_c2_seq1 | 1-aminocyclopropane-1-carboxylate oxidase 1-like | 0.2 (0.2) | 5.8 (1.6) | 0 | 43.1 | 190.4 | 103.7 | 0 | 551.5 | 35.5 | 10.5 |
| comp27248_c1_seq24 | uncharacterized protein LOC104449412 | 2.6 (1.4) | 3.5 (1.8) | 5 | 4.1 | 6.9 | 0 | 2.5 | 51.4 | 15.9 | 6.8 |

**$ For S.E. n=3**
