# Supplementary material for: RNA-sequencing based gene expression landscape of guava cv. Allahabad Safeda and comparative analysis to colored cultivars
Source: BMC Genomics. 2020 Jul 15;21:484. doi: 10.1186/s12864-020-06883-6 (PMC7364479; doi:10.1186/s12864-020-06883-6)
Supplement: Supplementary file 5 — Additional file 5: Table S5. Primer Sequences for candidate genes/internal control for qRT-PCR analysis. [file 12864_2020_6883_MOESM5_ESM.docx]

**TABLE S5. Primer Sequences for candiadte genes/internal control for qRT-PCR analysis.**

| **S.No.** | **Candidate Gene** | **Primer Name** | **Forward** | **Reverse** | **Amplicon Length in bp (Fwd Primer – Rev Primer)** |
| --- | --- | --- | --- | --- | --- |
|  | Phenyl-Ammonia-Lyase  (comp27773_c0_seq1) | PgASK31PAL_27773S1_qRT | CGAACGGGATCAAGGAATGC | CCTTGCCGAAGTCCTCTCCA | 117 (1733-1849) |
|  | Phytoene synthase  (comp27725_c0_seq1) | PgASK31PSY2_27725S1_qRT | CATCTCGAGCTCGGTGGTGA | TCCTCCGGCTTCTTCACGTC | 129 (666-794) |
|  | (R,S)-reticuline 7-O-methyltransferase-like (comp25759_c1_seq1,  comp25759_c1_seq5,  comp25759_c1_seq11) | PgASK31RML_25759S1_5_11_qRT | GGAGGGTTTCCTCGCTACAACA | CGCAAATCGAGCATACAACTCG | 130 (673-802; 978-1107; 631-760) |
|  | (R,S)-reticuline 7-O-methyltransferase-like  (comp25759_c1_seq4) | PgASK31RML_25759S4_qRT | GGGGGATGAAGATTGCATCAAG | TGACCAGATCGAGCACTGTTCC | 146 (265-410) |
|  | Glycerol-3-phosphate acyltransferase 5  (comp14631_c0_seq1) | PgASK31G3PAT_14631S1_qRT | CCGAGAGTCATGGTGGAGAGGT | TTAATGAAACCGGTGGCGTACC | 107 (504-610) |
|  | Peamaclein  (comp12564_c0_seq1) | PgASK31Peam_12564S1_qRT | TCCAATTTCCAGGCCGTAAGAA | TTGTGCCAATTCAATCCATTCG | 121 (940-1060) |
|  | CTP synthase-like  (comp22486_c0_seq10) | PgASK31CTPS_22486S10_qRT | GCATGCTTCTGTTGCTTGCAGT | ACGCAGCCTTGTAAGCTTCTGG | 106 (1620-1726) |
|  | Monodehydroascorbate chloroplastic  (comp26385_c2_seq73) | PgASK31MDAC_26385S73_qRT | ATTGAGTGCGCAGACCCATACA | TGGATCAAAGCTTCCGACATCA | 144 (1507-1651) |
|  | Probable 2-oxoglutarate-dependent dioxygenase AOP1  (comp26017_c0_seq1) | PgASK31AOP1_26017S1_qRT | TGTTCAGGATGGTCTGCGAGAG | TTCAAGTCGGTGTCGAGGTCTG | 124 (509-632) |
|  | Methionine synthase  (comp23125_c0_seq1) | PgASK31MetS_23125S2_qRT | GGAGGTTTGATGCACCCTTGAG | CACGCAAATTGGCAACGAGTAA | 168 (466 – 633) |
|  | Secoisolariciresinol dehydrogenase  (comp28595_c0_seq1) | PgASK31SecD_28595S1_qRT | CTACGTCCACTGCGATGTCACC | ATCCCAGCGTTGTTGAACATGA | 105 (367-471) |
|  | BEL1-like homeodomain 1  (comp17046_c0_seq2) | PgASK31BelHD_17046S2_qRT | TCTCGACAAATTCGGCTTCCTC | GACGAGACATTGCTCGGGTTCT | 156 (1871-2027) |
|  | Aminocyclopropane-1-carboxylic acid oxidase (comp22451_c2_seq1) | PgASK31ACO_22451S1_qRT | GCGCTGATGCCATAATCTACCC | CTTGACTGCATGGCTTTCATGG | 171 (257-428) |
|  | PREDICTED: uncharacterized protein LOC104449412  (comp27248_c0_seq1) | PgASK31Unc_27248S24_qRT | TTGGATATCTGCCACGACCTCA | TCATCAGATGGATGACCCTGGA | 126 (4544-4669) |
|  | Histone 3 **(Internal Reference)**  (comp27670_c0_seq1) | *Pg*ASK31H3_27670S1_qRT | GGGCAATTTCACGGACAAGC | TGCCCGTAAGTCTGCCCCTA | 155 (977-1131) |
