# Supplementary figures and images for: RNA-sequencing based gene expression landscape of guava cv. Allahabad Safeda and comparative analysis to colored cultivars
Source: BMC Genomics. 2020 Jul 15;21:484. doi: 10.1186/s12864-020-06883-6 (PMC7364479; doi:10.1186/s12864-020-06883-6)

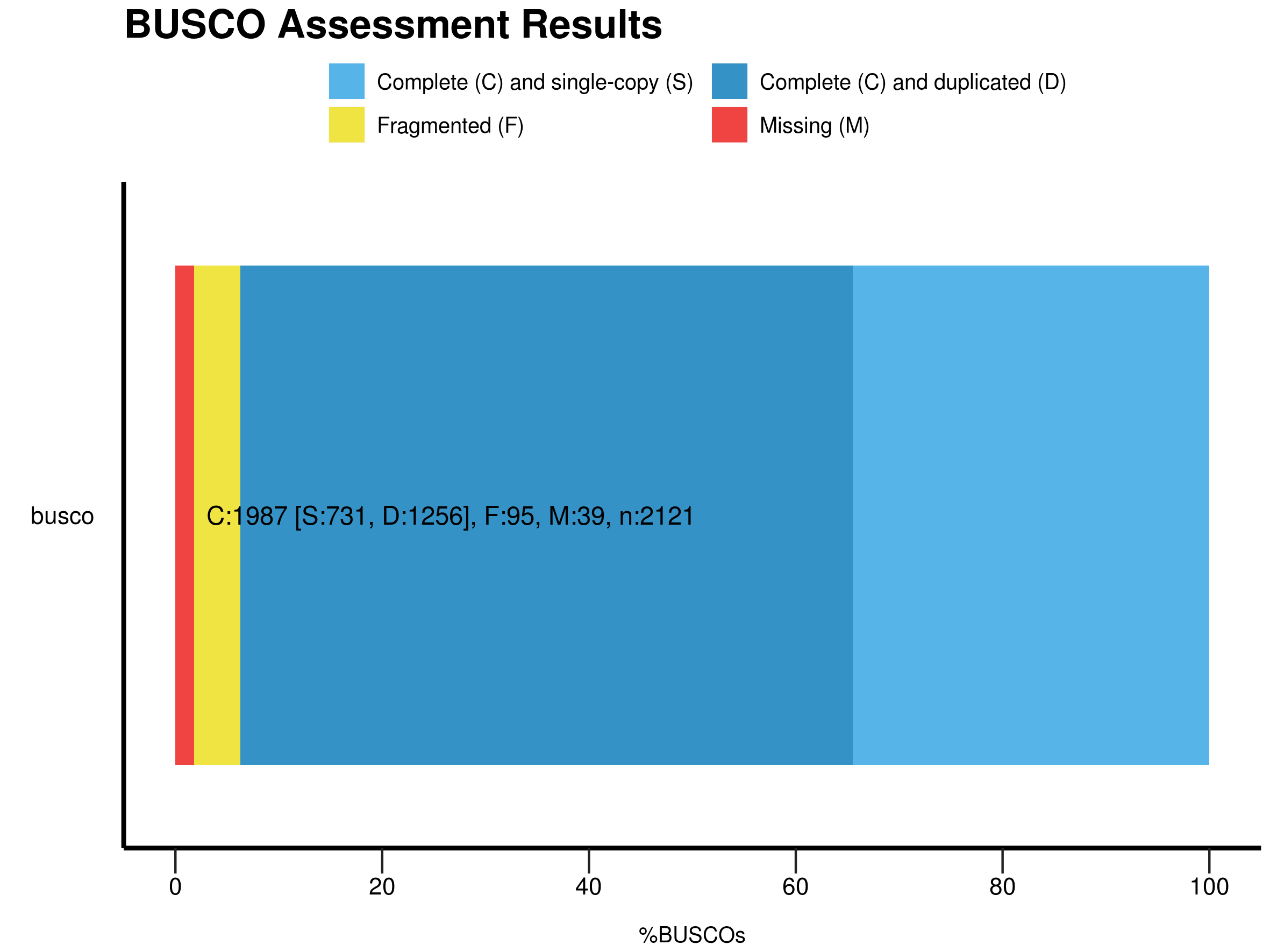

Supplement: Supplementary file 6 — Additional file 6: Figure S1. Summary of conserved orthologous genes (BUSCO) in the assembled guava transcriptome. [file 12864_2020_6883_MOESM6_ESM.png]

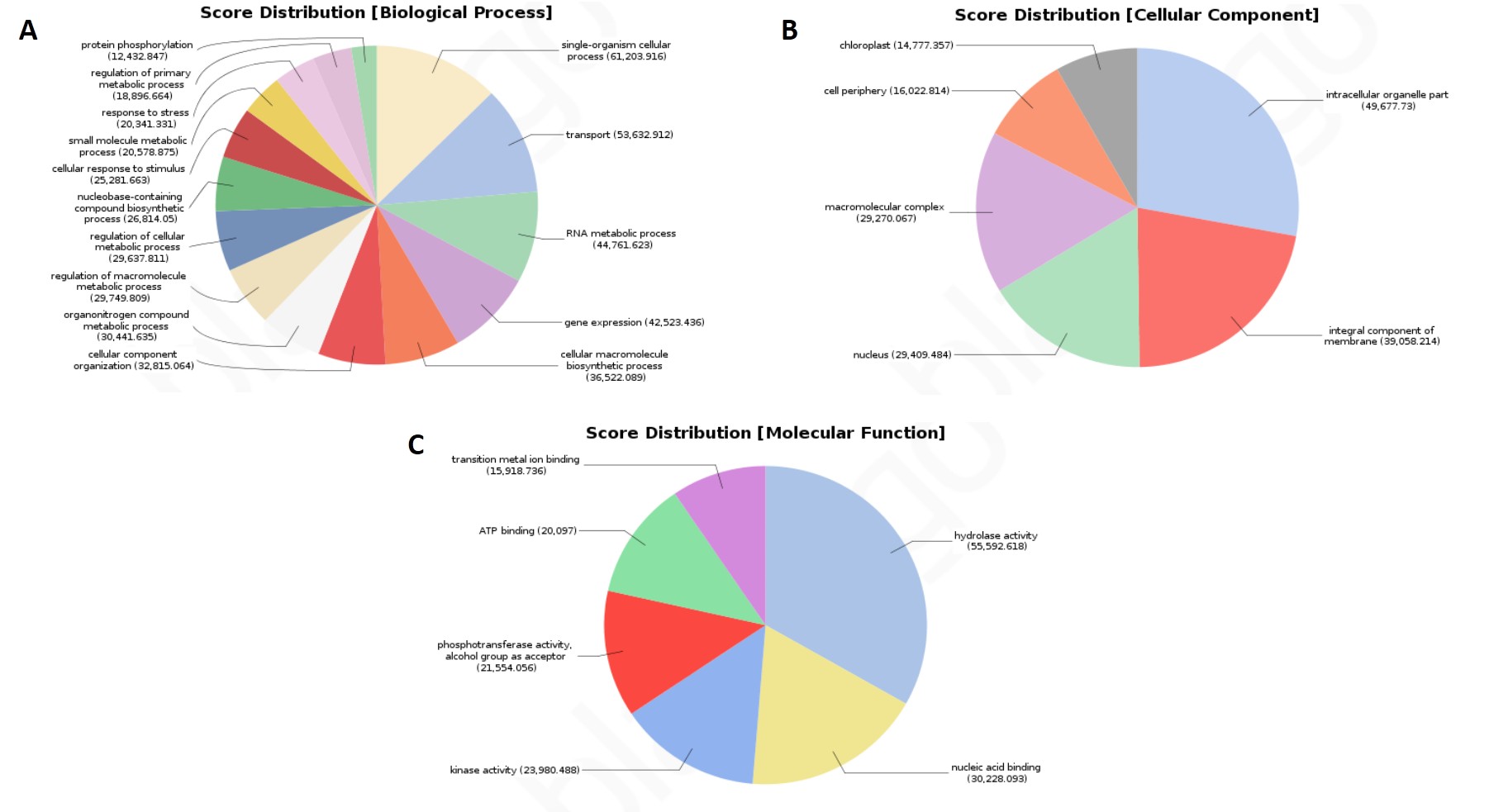

Supplement: Supplementary file 7 — Additional file 7: Figure S2. Blast2GO distribution of assembled transcripts into A) Biological processes B) Cellular component C) Molecular function. [file 12864_2020_6883_MOESM7_ESM.jpg]

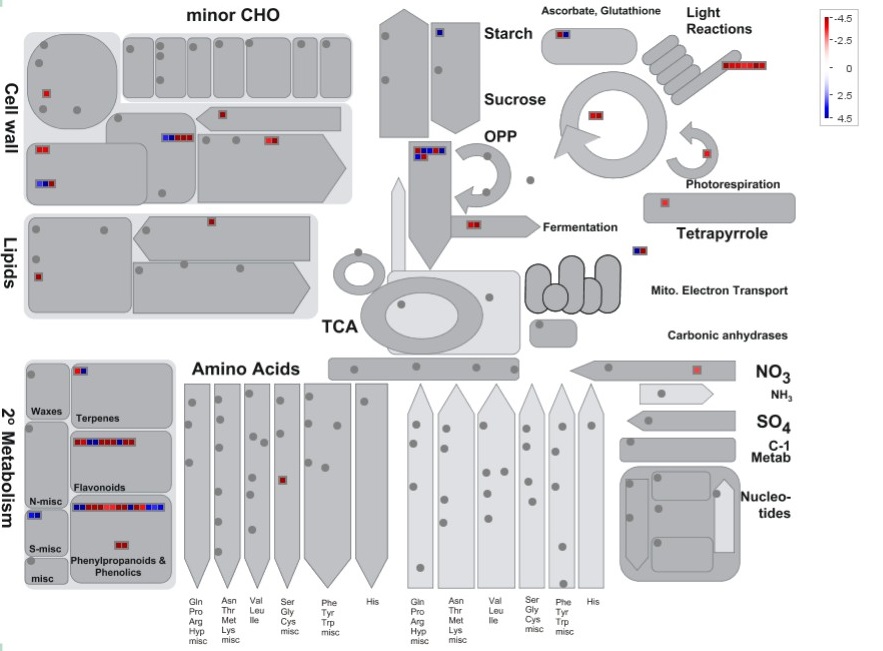

Supplement: Supplementary file 8 — Additional file 8: Figure S3. Metabolic overview with MAPMAN analysis of differentially expressed transcripts of mature fruit (0DF) vs Immature fruit (ImF) of cv. Allahabad Safeda. Up- and Down- regulated DETs are represented with blue and red squares, respectively with log2 transformed values. [file 12864_2020_6883_MOESM8_ESM.jpg]

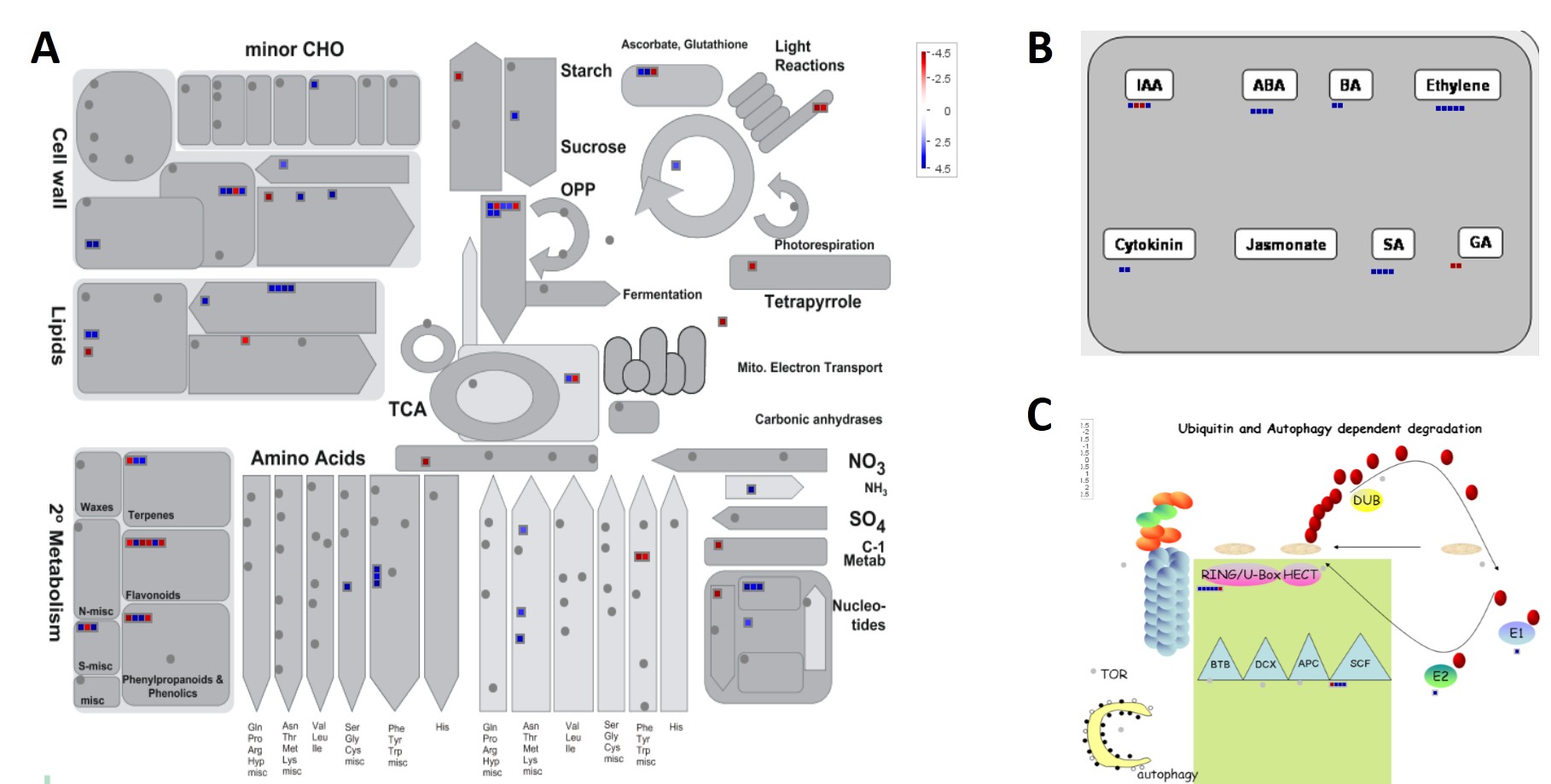

Supplement: Supplementary file 9 — Additional file 9: Figure S4. MAPMAN analysis of differentially expressed transcripts of ripe fruit (3DF) vs mature fruit (0DF) of cv. Allahabad Safeda A) Metabolic Overview B) part of regulation overview C) proteasome and autophagy. Up- and Down- regulated DETs are represented with blue and red squares, respectively with log2 transformed values. [file 12864_2020_6883_MOESM9_ESM.jpg]

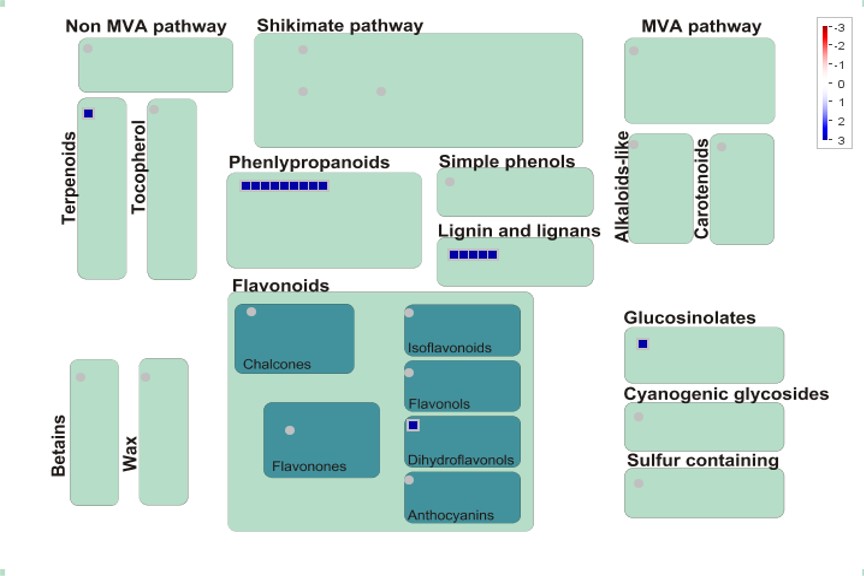

Supplement: Supplementary file 10 — Additional file 10: Figure S5. MAPMAN analysis of differentially expressed transcripts of apple color skin vs green skin of Apple Color – CISH-G5, shows up-regulation of the secondary metabolism pathway. Up- and Down- regulated DETs are represented with blue and red squares, respectively with log2 transformed values. [file 12864_2020_6883_MOESM10_ESM.jpg]

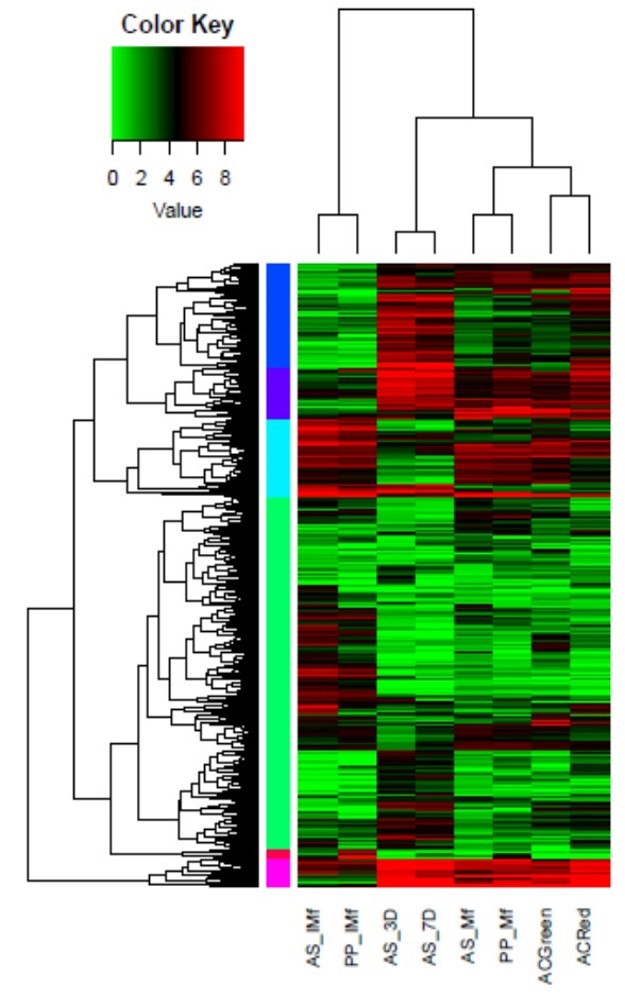

Supplement: Supplementary file 11 — Additional file 11 : Figure S6. Cluster analysis of differentially expressed transcripts among fruit stages of Allahabad Safeda (AS), Apple Color (AC) and Punjab Pink (PP) immature fruit (ImF), mature fruit (MF), 3 days after harvesting (3DF), 7 days after harvesting (7DF), green peel and red peel. [file 12864_2020_6883_MOESM11_ESM.jpg]

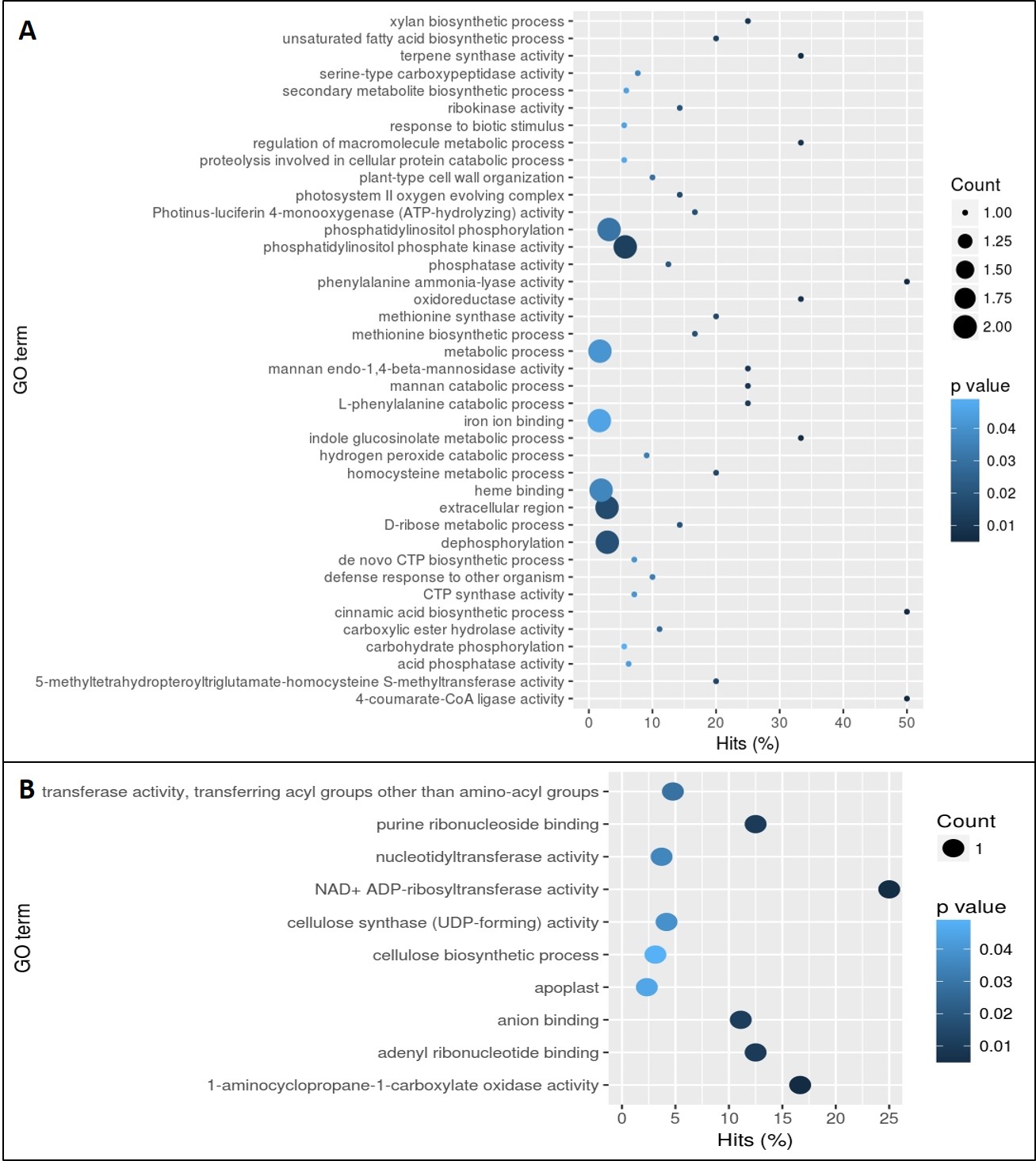

Supplement: Supplementary file 12 — Additional file 12: Figure S7. Gene Ontology enrichments between A) red and green peel of Apple Color B) mature fruit of Punjab Pink and Allahabad Safeda. [file 12864_2020_6883_MOESM12_ESM.jpg]
